# Moral Judgments of In-Group and Out-Group Harm in Post-conflict Urban and Rural Croatian Communities

**DOI:** 10.3389/fpsyg.2018.00212

**Published:** 2018-02-23

**Authors:** Michael A. Moncrieff, Pierre Lienard

**Affiliations:** The SEC Lab, Department of Anthropology, University of Nevada, Las Vegas, NV, United States

**Keywords:** morality, coordination, ethnicity, relational mobility, urban-rural, in-/out-group

## Abstract

Our research brings to light features of the social world that impact moral judgments and how they do so. The moral vignette data presented were collected in rural and urban Croatian communities that were involved to varying degrees in the Croatian Homeland War. We argue that rapid shifts in moral accommodations during periods of violent social strife can be explained by considering the role that coordination and social agents' ability to reconfigure their social network (i.e., relational mobility) play in moral reasoning. Social agents coordinate on (moral) norms, a general attitude which broadly facilitates cooperation, and makes possible the collective enforcement of compliance. During social strife interested parties recalibrate their determination of others' moral standing and recast their established moral circle, in accordance with their new or prevailing social investments. To that extent, social coordination—and its particular promoters, inhibitors, and determinants—effects significant changes in individuals' ranking of moral priorities. Results indicate that rural participants evaluate the harmful actions of third parties more harshly than urban participants. Coordination mediates that relationship between social environment and moral judgment. Coordination also matters more for the moral evaluation of the harmful actions of moral scenarios involving characters belonging to different social units than for scenarios involving characters belonging to the same group. Participants high in relational mobility—that ability to recompose one's social network—moralize similarly wrongdoings perpetrated by both in- and out-group members. Those low in relational mobility differentiate when an out-group member causes the harm. Additionally, perceptions of third-party guilt are also affected by specifics of the social environment. Overall, we find that social coordination and relational mobility affect moral reasoning more so than ethnic commitment.

## Introduction

Yugoslavia was an institutionally and economically integrated nation for decades (Hardin, 1997; Oberschall, 2000). It quickly disintegrated along ethnic lines once civil unrest started in the first half of the 1990s. Yugoslavia is an illustrative example of a salient type of violent and abrupt nation breakdown into smaller antagonistic units, often ethnically marked, following an extended period of tranquility (Horowitz, 1985). For more than 40 years, relations between the two major population segments, the Croats and the Serbs, were peaceful and cooperative in the area that would become the modern-day Republic of Croatia following the Homeland War (1991–1995). Mixed marriages were common and Serbs and Croats saw each other first as friends, colleagues, and co-workers, paying little attention to each other's ethnic background (e.g., Glenny, 1996; Hardin, 1997; Oberschall, 2000; Sekulić et al., 2002). Public opinion research before the war indicated that over 85% of the Yugoslav population thought that neighborhood relationships were good or satisfactory and <7% believed that the nation would eventually fall apart (Cohen, 1993, p. 173).

A shift in ethnic sentiments seems to have occurred immediately before the breakdown of inter-ethnic communal life in Croatia (e.g., Corkalo Biruski et al., 2004). Distrust between communities quickly arose (Ajdukovic and Corkalo Biruski, 2004) and community interactions escalated into violent outbursts (Čorkalo Biruški, 2012). Such situations prevailed particularly in rural locations (Allcock, 2002). As elsewhere in former Yugoslavia, the urban-rural divide in the region that is now included in the Republic of Croatia seems to have been central to stirring up social strife (Petersen, 2002). Weidmann (2011) notes that the primary areas of mobilization at the onset of the general conflict were in regions with a rural character. Brubaker (1995, p. 123) also observes that the mobilization against a national Croatian aspiration was greatest in Serbian rural communities. Furthermore, although the first major attacks in many communities throughout Yugoslavia were initiated by insurgent paramilitary units (Čorkalo Biruški, 2012), as the conflict proceeded, violence between neighbors, friends or long-known others became more the norm (Bringa, 1995; Petersen, 2002). Once familiar community members were now no longer thought of as moral beings (Bringa, 1995; Petersen, 2002). The reciprocal violence between Croats and Serbs was particularly pronounced in the central rural Krajina conflict region of Croatia (e.g., Stevanović, 1998; Grandits and Carolin, 2003). By the end of the war in Croatia an estimated 37,000 had been wounded, 13,583 killed, and 700,000 people had been displaced (Perkovic and Puljiz, 2001). What were the specific features of those social environments affected during the war that could explain the fast descent into such widespread brutality and chaos?

The onset of the breakdown of Yugoslavia saw the realignment of several population segments around competing moral projects, which led individuals to redraw *en masse* the limits of their moral circle (Denich, 1993; Brubaker, 1995, p. 56–57). The concept of moral project encapsulates three major aspects: social coordination, monitoring, and sanctioning. A moral project first concerns a community's agreement on and commitment to actions deemed acceptable in the pursuit of social and political life (Boehm, 2000, p. 80). Thus, coordination on such set of rules reduces social transaction costs. It reduces uncertainty in social dealings by allowing agents to expect non-randomness in the behavior of others and to act on the base of such knowledge. Non-compliance does the opposite; it re-injects a level of uncertainty that raises the risk of miscoordination between social agents, hence the costs of socially transacting. Individuals are thus inherently incentivized to monitor the behavior of others for compliance given the risks that deviance brings. Members of moral communities monitor for norm violations and may attempt to muster support to sanction their perpetrators through, for instance, the sharing of gossip, by publically shaming the violator or through other more radical means. The ability to coordinate with others to punish violators often helps to resolve conflict quickly and eliminates the need for further escalation (DeScioli and Kurzban, 2013).

For a moral project to succeed, it requires establishing social boundaries and excluding non-supporters. Moral communities can eventually amount to political coalitions searching to assert power in an effort to manipulate or eliminate deviants (Boehm, 2000). Supporters of a moral project can recruit others by broadcasting information about rivals' behaviors that would conflict with deeply held values and norms. The information evokes moral outrage and motivates individuals toward action (Jasper, 1998). Rumor of immoral acts perpetrated by opposition members are often highly effective tools of mobilization (Horowitz and Varshney, 2003; Bhavnani et al., 2009). During the breakup of Yugoslavia, Serbian and Croatian news stations aired identical images of victims of atrocities, attributing the responsibility of the wrongdoing to their respective opponent (Milosevic, 1997, p. 119). Accusations of rape by members of the opposing group were also common practices (e.g., Cohen, 1998, p. 222; Markovic, 1998, p. 323). Moral suasion, the use of morality to influence the behavior of others through appeals to “*do the right thing*,” may also be effective in motivating individuals to align with the objectives of a moral project, particularly when employed in conjunction with punishment (Dal Bó and Dal Bó, 2014). As noted by MacDonald (2010: 7) moral arguments, a prominent feature of propaganda, focused on the wrongdoing of the foe while considering one's own group's actions to be morally justified.

During periods of tense confrontation between competing social units, actions may be reassigned alternative value in response to the emerging individual's duty to his/her coalition (i.e., it is *right* to defend my people, it is *wrong* for the other coalition to harm us). Before the war, taking a neighbor's property would have been entirely unacceptable. It became less reproachable during the war if it was in support of one's coalition (e.g., Leutloff-Grandits, 2006). During the breakdown of Yugoslavia, individuals had to withdraw from supporting their inter-ethnic social networks to invest more intensively in their primary ethnic affiliations (Čorkalo Biruški, 2012). Acting as a moral individual in the eyes of ethnic fellows eventually required affirmative actions in support of one's group (see for example, Human Rights Watch, 1993: 79; Stigelmayer, 1994: 156–7). Optimal actions should ideally be zero-sum, i.e., they should combine benefits for one's group and costs to outsiders, which eventually would lower the threat that the latter represents. The new demands associated with the strengthening of one's ethnic affiliation may explain why sudden shifts in acceptable moral behavior occur during periods of ethnic conflict. The reshuffling of moral priorities should be particularly striking in plurinational populations relying heavily upon informal social institutions (Hardin, 1997).

We propose to investigate aspects of these dramatic shifts in moral accommodations that lead to the dramatic realignment of social networks and moral circles along ethnic lines, using a traditional moral vignette technique. More specifically, we assess how features of the social environment, such as perceived coordination and social agents' ability to rearrange their social network (i.e., relational mobility), affect moral reasoning and judgment in small and large communities of the Republic of Croatia that have relatively recently experienced varying degrees of exposure to conflicts. We argue that the observed variations in moral reasoning and judgment are in part best understood as the result of processes of coordination, more so than as the result of commitment *per se* (i.e., affective attachment, investment, or ideology) to a specific group identity.

## Background

### Coordination and collective action

Humans regularly engage in collective action and have so throughout history (Ostrom, 1990; Price et al., 2002; Tooby et al., 2006). Collective situations of cooperation where three or more individuals interact are known as *n-*person exchanges (e.g., Von Neumann and Morgenstern, 1944, 2007; Nash, 1950). If successful, collective actions provide significant benefits e.g., defense against rival coalitions (Chagnon, 1983), increased status and access to mates (Bissonnette et al., 2015), and protection of limited resources (McCay and Acheson, 1987). For an *n*-person exchange to constitute an evolutionarily stable strategy, the benefits reaped by individuals must be, on average, proportional to their contribution to the joint action (Trivers, 1971; Hamilton and Axelrod, 1981; Smith, 1982; Axelrod, 1984). When acting collectively, social agents must address the essential challenge of coordination and the threat of exploitation (Tooby et al., 2006). Exploitation occurs when individuals obtain benefits from the collective action while not contributing their fair share. For our research, we focus solely on the challenge of coordination (Olson, 1965; Ostrom, 1998; Price et al., 2002).

### Coordination and mutual knowledge

In the *Battle of the Sexes*, Luce and Raiffa (1957) have formalized the problem that two agents face when they wish to coordinate in a situation of incomplete information. A couple, say, Marcia and Chris, decides to meet the next evening after work to go to an event together. The decision of the specific venue where the couple would go is not finalized at the time of making the original decision to go out. The next day they are not able to communicate with each other. The couple thought of two options: cinema or bowling. Marcia would pick bowling over cinema, while Chris much prefers cinema. They both look forward to being together, rather than spending time separated were they to fail at coordinating their respective choice of venue. Marcia's and Chris' decision are in a relation of codependency. If both Marcia and Chris want to please each other, they will both fail to meet and will spend the evening separated. If both decide to follow their preference, they will once again fail to meet. A solution to the problem could be that, Marcia knowing that Chris is rather selfish and Chris knowing that Marcia knows that, they both go to the cinema. Or alternatively, Chris knowing that Marcia would be extremely disappointed if he were to selfishly select the cinema and Marcia knowing that Chris knows her very well, they both go to the bowling alley.

As the number of people grows, the assessment of the likelihood of success of a collective action is made even more complex than in *The Battle of the Sexes*, as more participants means more potentially diverging interests, or competing incentives. To coordinate, individuals must be incentivized by the potential benefits of participation and have a common knowledge of some of the specifics of the collective action to be performed and the associated potential payoffs (Schelling, 1960). Individuals engaged in the interaction must know X, and that the others also know X, and all know that the others know X and so on (Thomas et al., 2014). Individuals are much more willing to engage in collective action, especially risky ones, when common knowledge is achieved (Thomas et al., 2014). If Chris has something to apologize for, and Marcia knows that, Chris knows it, and Chris knows that Marcia knows it, the couple will more certainly meet at the bowling alley, the benefit to both Chris and Marcia outweighing the cost to Chris. But John wanted to come along…

### Morality as a solution

Relevant aspects of our moral psychology provide solutions to complex computational problems associated with multiple-partner exchanges, such as the estimation of the threat that competing interests might yield and the reckoning of the individuals' motivation to act in ways that would maximize the collective outcome (Tooby et al., 2006). In their pursuit of ordinary goals, social agents regularly enter into competition with each other. Such rivalry has the potential to destroy mutually beneficial social transactions, entailing significant costs, were miscoordination to lead to a complete failure of cooperation, to the loss of social partners or resources, and conceivably, to a violent opposition. Mutually beneficial exchanges are advanced when individuals refrain from a compulsive realization of immediate self-interests. In peaceful and efficient social worlds, forbearance is widespread. Individuals routinely give up the benefits that would be afforded by violating a moral norm, so long as others in their community do the same (Tooby et al., 2006). Indeed, individuals very much mind other people's behavior. Is their behavior disrupting the prevailing arrangements? The stakes are high—as cooperation might quickly unravel into a situation of too great uncertainty—particularly so in times of conflict when coordination and cooperation are vital for safety and security (Tooby and Cosmides, 1988).

Compelling social institutions, in the forms of concerted norms, values, and other formal and informal social “charters,” often in combination with powerful adjuvants such as moral emotions and reputational concerns (Sperber and Baumard, 2012), contribute to alleviating collective action problems, i.e., situations where joint actions for common goals, though potentially beneficial, are hard to achieve given particular disincentives. Moral emotions, such as guilt and shame, likely evolved to motivate behaviors that reduce the likelihood and costs of being socially exposed violating a moral rule (Sznycer et al., 2015). In this way, moral emotions can be conceived of as commitment devices encouraging individuals to forego immediate self-interests for longer-term *moral* strategies (Frank, 2004; italics added). Indeed, Sznycer et al. (2012) found that proneness to shame increases in environments where the cost of being sanctioned for a violation is great. The moral emotion of guilt is triggered when one defects from cooperative norms and, eventually, may motivate individuals to make amends for their behavior (Gibbard, 1992; Baumeister et al., 1994; p. 138; Ortony et al., 1988; Leith and Baumeister, 1998; Tangney and Dearing, 2002). Given their functional role in maintaining moral coordination, the proneness to feel guilty should also be sensitive to the dynamics of a social environment.

Effective coordination plays an essential role in moral condemnation (DeScioli and Kurzban, 2009, 2013; DeScioli et al., 2011). Single-handedly moralizing others' behavior comes with risk, e.g., of withdrawal of social partners, retaliation, or ostracism. Given those perils, it is common for individuals to attempt to associate with others for condemning perceived deviants, hence assuring that the burden of sanctioning is borne by a collective (Boehm, 1993). The association of agents to condemn deviants falls into the category of typical *n*-person exchange (Tooby et al., 2006).

### Diversity of moral project content

The moral principles of fairness, equity, justice, decency, and duty are universal (e.g., Banerjee et al., 2010; Graham et al., 2013). Specifics of moral projects do certainly vary across socio-cultural units. Previous research has shown that moralizing differs cross-culturally as well as by individual variables such as social class and age, although the reasons for these differences are still debated (e.g., Shweder et al., 1997; Haidt and Joseph, 2004; Rai and Fiske, 2011; Haidt, 2012). Social environments may differ in how individuals process moral attributions, the attribution of a level of morality on the basis of the assessment of observable behavior. An and Trafimow have shown that moral attribution varies between cultures deemed individualistic and the ones considered as collectivist (An and Trafimow, 2014; An et al., 2016). Others, still, have studied the impact of social structures on moral responsibilities, hence the likelihood of finding much variation in moral reasoning across the globe (Abarbanell and Hauser, 2010).

Such culture-specific content may be solutions to coordination challenges that arise in particular social environments. As information about the harm of cigarette smoking increased, the judgment of the behavior went from a non-issue to a sensitive moral question with multiple ramifications (e.g., healthcare cost, second-hand smoking, harm to fetus) in the United States (Rozin and Singh, 1999). Increased knowledge of the risk of smoking led to public debates about second-hand exposure. Non-smoking advocates rallied around new mores that restricted the actions of smokers to enclosed environments away from others. Fluctuations in moral debate and condemnation can best be grasped when we understand that moral norms act as coordination devices. Through a progressive coordination on an emerging moral project, moral militants can become efficient at restricting undesired third-party behaviors.

### Moral recruitment and social conflicts

The coordinational nature of moral norms makes them appropriate candidates to address novel social conflicts. Arbitrary moral rules can become coordination points when they support coalitional goals, such as the lowering of status of rival groups (Tooby and Cosmides, 2010). The emergence of the apartheid system in South Africa constitutes a good example of the recruitment of moralization to maximize the success of a particular community. Afrikaners' apprehension of economic and political competition from blacks led to the formation of morally enforced social boundaries, and eventually to the establishment of the apartheid system (Brits, 2000). Upon settling the land, the European communities that were to become the Afrikaners bolstered boundaries between racial groups by a widespread moral objection to mixed relations and inter-marriage (Freund, 1976; Malherbe, 2010). In the competitive interethnic context of South Africa, racial ambiguities, such as when a child with a black-African phenotype was born to Afrikaner parents, often led to conflicts (see Stone, 2008). Progressively, official legislations were drafted to explicitly address such matters and clarified the categorization of individuals on the basis of their appearance or ancestry (Johnson, 1989; Hyslop, 1995). Despite their *ethical rationalistic* immorality, the *moral* norms enforcing separation of racial communities eased the resolution of internal conflicts, maintained cohesion, and, in definitive, assured Afrikaners' control of the segregation system.

It is relatively easy for small and organized communities and militant segments of larger populations, such as the South African Afrikaners, to muster support for emerging moral projects. Members of militant social units are more likely to share common goals and to have fewer diverging interests than members of larger communities. The redundancy, imbrication and overlapping of social networks and the concomitant sense of social obligations and duties to other community members insure social cohesion, expediting the process of coordination on emerging moral rules. Moral rhetoric, too often disconnected from positions that would be held by ethical rationalists, is commonly used to recruit individuals and mobilize social factions during episodes of social strife (e.g., Spencer, 1990, 1992; Espeland, 2007; Kiernan, 2007). An edifying example would be the National Socialist Party's depiction of the customs, actions and, eventually, nature of Jews as immoral, during its ascension to power (Hinton, 2002). The role of coordination in moral reasoning may also explain why newly-strengthened coalitional affiliations often end up trumping preexisting moral accommodations during periods of conflict, leading to the perpetration of atrocities (e.g., Horowitz, 1985; Hardin, 1997; Halpern and Kideckel, 2000; Waller, 2002; Hatzfeld, 2005; Espeland, 2007).

### Cosmopolitan vs. sectarian moral universe

The modern urban societies in which much of the world's population dwells today are vastly different from the small communities that shaped much of deep human history. Small communities—within which social transactions are typically repeated, members have extensive knowledge of each other, and informal institutions guarantee the rewarding of appropriate behavior or the sanctioning of deviance—typically differ from larger counterparts in the frequency of unrepeated (*a.k.a*., one-shot) social interactions, the degree of achievable anonymity, and the level of reliance on formal compliance-monitoring institutions (e.g., Amato, 1993; Yamagishi and Cook, 1993; Hardin, 2001). Individuals in small communities rely on coextensive, co-dependent, and mutually-reinforcing networks locating their respective social reason in distinct life domains, whereas social agents in more open social worlds partake in multiple, distinct, and limited-in-scope networks, each with its independent set of bonds based on, e.g., friendship, work occupation, or choice of leisure (Cook and Hardin, 2001). The perceived ability to establish novel relationships in one's social world, otherwise known as *relational mobility*, should necessarily be lower in communities where network imbrication is more pronounced.

We choose to focus on communities on either side of the rural-urban divide, as we think that it is the best way to access populations that have the respective profile of either a small or a larger social world with their respective modality of coordination and opportunities of relational mobility in a modern context. Given the dramatic events that occurred during and immediately after the war, such as ethnic cleansing and massive migratory fluxes, the distinction between native urban or rural individuals has probably been blurred to some extent. Note also that the rural inhabitants of the selected area were on the frontline to a much greater extent than the urbanites during the Croatian Homeland War and are still being exposed to regular expressions of irredentism. The participants from that area should be more likely to frame the social world in coalitional terms.

## Croatian field site

The Republic of Croatia provides a remarkable field location to study the effects of features of the social environment on morality, given its recent history of ethnic conflicts. The patchwork of majority-minority ethnic groups, small-/large-scale living, and distinct levels of market integration allow for differentiating the factors impacting moral reasoning, evaluation, and judgment.

Zadar and Benkovac, two municipalities of the Dalmatian Coast of the Republic of Croatia in Zadar County, have experienced varying degrees of ethnic conflict during the Croatian War of Independence (1991–1995). The two neighboring municipalities (28 miles separate their respective main agglomeration), offer very distinct social and demographic conditions (see Table 1).

**Table 1:** Municipality demographics.

	**Zadar**	**Benkovac**
Mean Age	41	41
**EDUCATION**		
High School or Secondary	**58%**	**48%**
Elementary School	**15%**	**23%**
Uneducated	**1%**	**8%**
Mean Number Per Household	**2.71**	**3.04**
**RELATIONSHIP STATUS**		
Single	~30%	~30%
Married	~58%	~58%
Divorced or Widowed	~13%	~13%
Widows	14%	18%
Widowers	3%	4%
**SETTLEMENT TYPE**		
Urban	**95%**	**26%**
Rural	**5%**	**74%**
Living Since Birth in Municipality	**59%**	**74%**
Agricultural Land	**1%**	**40%**

*Interesting numerical differences between the municipalities are bolded. All reported data is from (Republic of Croatia Bureau of Statistics, 2011)*.

Zadar is an urban municipality with 95% of its residents living in urban areas, the rest of the population dwelling in rural/other settlements (Republic of Croatia Bureau of Statistics, 2011)^1^. Benkovac is a rural municipality with a low 26% of its residents residing in urbanized settlements, primarily in its main town, and 74% living in rural settlements. The average number of individuals per household is lower in Zadar (*M* = 2.71) than in Benkovac (*M* = 3.04). Most residents of Zadar (58%) have completed the high school level or a secondary education program, 15% have completed the elementary school level, and 1% is uneducated. The University of Zadar has ~6,000 students and over 620 staff members, thus contributing to the fact that 23% of Zadar residents have a degree from a college or university. Of the residents of Benkovac, 48% have completed the high school level or a secondary education program, 23% have completed the elementary school level, 8% are uneducated, and 6% have a college or university education. Zadar and Benkovac share similar mean age (41 years old) and marriage demographics (~ 30% single, 58% married, 13% divorced or widowed). Following the violence of the war, a large number of women (18% Benkovac, 14% Zadar), and a smaller number of men (4% Benkovac, 3% Zadar) are registered as widows and widowers. For the Republic of Croatia, 67% have remained in the municipality in which they were born. In Benkovac 74% (constituting 62% of all Benkovac residents) and in Zadar 59% (constituting 53% of all Zadar residents) have remained all their life where they were born. This illustrates the local history of enduring interactions between a majority of the residents of the study areas. Our research participants are inhabitants of either one of those two municipalities and their respective hinterland.

In Zadar, retail is the largest sector of the economy with 23% of the employment, followed by education (12%) and public administration (11%) (Republic of Croatia Bureau of Statistics, 2011). Benkovac's inhabitants are primarily involved in manufacturing (22%), retail (17%), and public administration (11%). Only 1% of households in Zadar own agricultural land (*M* = 0.17 acre/home), compared to 40% of households in Benkovac (*M* = 2 acre/home).

**Figure 1:**
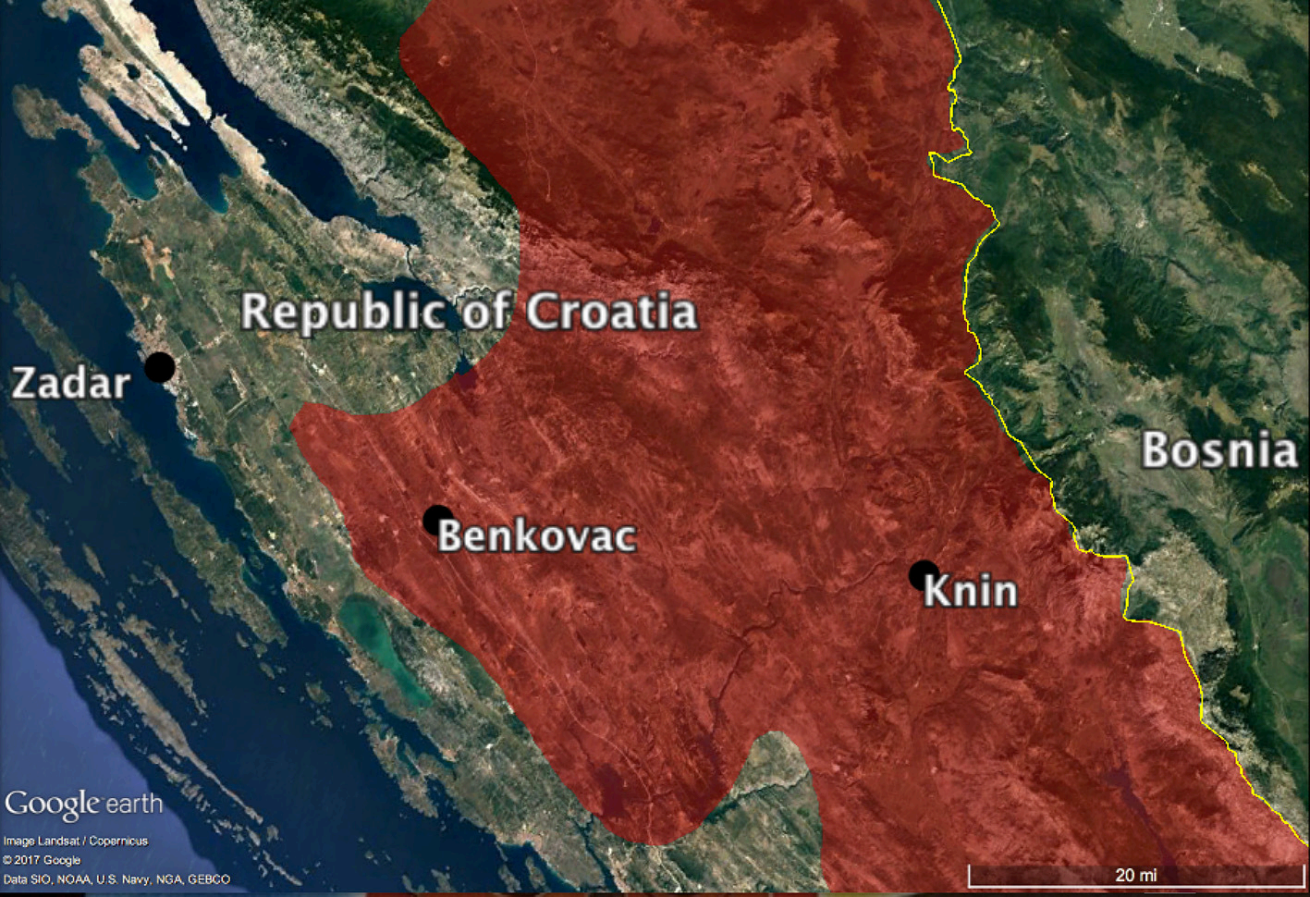
Map of the research region in the Republic of Croatia. War-time Serb-occupied areas are in red.

Inter-ethnic relations in Benkovac played a pivotal role in the events leading up to the declaration of independence of the Republic of Croatia and the eventual breakup of the Republic of Yugoslavia (Table 2). Before the war, Serbs made up the majority of the population in Benkovac at 57%, against a Croat population of ~41% (Tanner, 2001). Croats held only 18% of the employment positions in the local government, a discrepancy compounding the then-prevailing interethnic tensions (Tanner, 2001). After the Republic of Croatia's declaration of independence in 1991, Benkovac experienced rioting and was among several locations where the first armed aggressions occurred in Croatia (Tanner, 2001, p. 233). Benkovac and its hinterland saw the intensive involvement of their population in the Croatian Homeland War and for a while came under full Serb control (see Figure 1). Following the Croatian offensive battle *Operation Storm* in 1995, Serbs were expelled from the municipality and its surrounding villages. *Operation Storm* left 1,300 civilian homes damaged in 14 Benkovac villages and 130 homes in three villages in Zadar's hinterland (Klajn, 2007, p. 271). By 2010, aggressions against Serbs had largely diminished in most parts of Croatia but were still pronounced in the region of Benkovac (Human Rights Watch, 2006; US Department of State, 2010, p. 1242).

**Table 2:** Pre-War and Post-War Ethnic Compositions by Municipality.

	**Pre-War**			**Post-War**		
	**Croat (%)**	**Serb (%)**	**Other (%)**	**Croat (%)**	**Serb (%)**	**Other (%)**
Zadar	83	10	7	94	3	2
Benkovac	41	57	2	85	14	1

The populations in the town of Benkovac of 11,026 inhabitants is now 84.9% Croats, 13.8% Serbs, and 1.3% “others” (Republic of Croatia Bureau of Statistics, 2011). Thirty-nine villages surround the town of Benkovac ranging in population from 13 to ca. 530 residents. The majority of these villages are inhabited by Croats.

Zadar's population was less directly involved in the war. Before 1991, its population was 83% Croat and 10% Serb (Republic of Croatia Bureau of Statistics, 1991). Its metropolitan area currently has 75,062 residents with Croats making up 94.2%, Serbs, 2.9%, and “others,” 2.1% of the population (Republic of Croatia Bureau of Statistics, 2011).

## Hypotheses

Specifics of the social environment affect moral condemnation; coordination mediates it. Societies with small population size permit face-to-face interaction and extensive knowledge of co-residents. The choice of social networks is reduced. Increased common knowledge is also a striking feature of such social environments; individuals share mutual expectations about each other's behavior, attitudes, and dispositions, which greatly facilitate partner selection and social coordination. Large-scale social worlds involve greater amounts of one-shot interactions where individuals have little knowledge of each other. The choice between social networks and affiliations is greater than in a small community; it should therefore be easier to shift affiliations by joining other competing networks. Common knowledge is reduced to those within one's immediate social networks, and individuals are unlikely to have strong expectations about the behavior of unassociated others within their community. The characteristic features of small communities are to some extent less likely to condition the interaction of agents in the wider social world.

We conducted an experimental survey research with participants in the regions of Zadar and Benkovac to study how distinct social contexts differentially impact moral evaluations. Populations at these locations live in *more or less* open social networks, afford *more or less* relational mobility, and are *more or less* coordinated. Moral cooperation being a form of *n*-person exchange, we can make the following predictions about the impact of the social environment on moral judgments:

H_1_: Coordination predicts the degree to which individuals will morally condemn harmful actions.
  H_1.1_: *Participants highly coordinated with others evaluate harmful actions more harshly than those lowly coordinated*. Indeed, given their positions in the social world, well-coordinated individuals should fear less the risks associated with moral condemnation, those risks being reduced as more individuals share the costs of condemnation. Conversely…
  H_1.2_: *Participants lowly coordinated with others evaluate harmful actions far less harshly than those who are highly coordinated*. Individual costs associated with moral condemnation (e.g., opportunity costs, threat of retaliation) are the highest for those individuals who lack support for condemning the immoral behavior of others.

H_2_: Relational mobility modulates moral judgment.
  H_2.1_: *Relational mobility will matter more when the moral wrongdoing involves members of different coalitions vs. members of the same coalition*.
  H_2.2_: *Participants who perceive themselves to be high in relational mobility evaluate moral wrongdoing between two coalition members more harshly than those who are lower in relational mobility*. Individuals who are more mobile deal with a broader social world, for these individuals it is more important to have a world not divided by in-group and out-group concerns.

H_3_: *Living in a rural environment modulates ethnic commitment*.
  Participants from the central Croatian rural environment are likely to express stronger ethnic commitment given their situation at the forefront of the ethnic conflict during the Croatia Homeland War. Living in this region increased the likelihood of personal exposure to ethnic violence during the war. Inhabitants of Benkovac and its hinterland currently experience greater exposure to out-group (i.e., Serb) coalition members and reminders of ethnic tension than city dwellers in Zadar.

We did not have a set of very strong predictions for one of our measures, third-party guilt. The following hypothesis was exploratory only.

H_4_: *The scale of the social environment affects perceptions of third-party guilt*.
  H_4.1_: *Participants from rural areas who harshly judge outgroup third-party misbehavior will be less likely to say that the out-group member will experience guilt for their actions*. Given the more constrained social world found in rural environments, participants should be particularly sensitive to an out-group third-parties' actions when evaluating their moral nature given the higher cost and lower benefit of including potentially immoral agents in their social world.
  H_4.2_: *Participants from urban areas who are highly mobile should not categorize differently the pro-social or anti-social propensity of in- and out-group members*. Highly mobile urbanites (more cosmopolitan) rely on larger social and professional networks, which should disengage their cognition from a coalitional positioning.

## Method

### Participants

We recruited participants from the urban municipality of Zadar and the rural communities of Benkovac and surrounding villages in Zadar County. Participants (*n* = 30) were first recruited through a systematic door-to-door method. Census enumeration districts in Zadar and Benkovac were randomly selected and researchers approached the inhabitants of every third residence starting at each enumeration district's geographic boundary. When there was no answer, the researchers attempted to revisit the home once more on another day. Because of a low response rate (*n* = 30), additional participants (*n* = 80) were recruited using a snowball method that began with local informants recommending initial participants. We continued to recruit additional participants upon recommendation from others in Zadar (for a total *n* = 51), Benkovac (for a total *n* = 31), and the surrounding villages (for a total *n* = 28). Upon completion of the survey, participants were offered 20 Kuna (~$2.94) and thanked for their time and participation. The University of Nevada Las Vegas' institutional review board approved the research (#885040-1). Five participants were excluded for not passing the reading comprehension check.

The majority of respondents in our sample self-identified as Croat (*n* = 103), compared to Serb (*n* = 1) or “other” (*n* = 1). Participants were exposed to the war at an age where they were able to have elaborate memories i.e., participants over the age of 30: 86% in the Benkovac area and 55% in Zadar were above that threshold. The samples from the urban and rural areas did not significantly differ in regard to age, sex, income level, or marital status. The whole sample included 45 men and 58 women with an average age of 38 (range = 18–89). Urban participants were more educated *X*^2^ (2, *N* = 102) = 42.35, *p* < 0.001. On average our rural participants had obtained a technical school education (~12 years), while urban participants had been through some professional studies (~14 years).

### Materials

#### Moral scenarios

We created four vignettes, each describing a situation where two social agents interact. One of those individuals acts in a wrongful manner toward the other one. The two agents either belong to the same socio-cultural group or to distinct ones.

Example (1)—individuals belong to the same socio-cultural unit (henceforth: within-coalition): A Chatic man is driving around completing his errands for the day and sees a Chatic man stuck on the side of the road. He does not help the man and continues driving.

Example (2)—individuals belong to distinct socio-cultural units (henceforth: without-coalition): A Lamak man is driving around completing his errands for the day and sees a Chatic man stuck on the side of the road. He does not help the man and continues driving.

The additional vignettes are in the Supplementary Materials. The scenarios are built around the following wrongful act: a man ignores another man stuck with his car on the side of the road; a man gets into an argument with a male neighbor and punches him; a woman spreads a rumor about her neighbors stealing from others in the community while she is unsure whether the rumor is true; and a man finds the wallet of another man on the street with money in it and decides to keep the money. We included four filler vignettes for computations of the coordination variable (see below) those vignettes involved the wrongdoing of a single individual: a youngster who smokes marijuana; a man who commits suicide by jumping from a bridge; a woman who drinks too much and injures herself because of it; and a man who gambles away the last of his personal savings (see supplementary materials). For each vignette, participants were asked to evaluate the action (How good or bad is what [the protagonist] did?) on a 1 (extremely bad) to 7 (extremely good) scale. All responses were reversed scored.

Each participant's *within-coalition moral evaluation* score is computed as the average of his ratings for the two scenarios involving characters from the same socio-cultural unit. Each participant's *without-coalition moral evaluation* is computed as the average of his ratings for the two scenarios involving characters from distinct socio-cultural units. ANOVA showed no statistically significant differences between the average ratings for the different scenarios.

#### Moral coordination measure

We operationalize *moral coordination* as the absolute difference between the four filler moral vignettes to compute the participant's rating of the moral scenario minus their second rating which is the evaluation of other people's likely rating of the character's behavior. The four filler vignettes are only used to compute the moral coordination score. As previously introduced, when people have an expectation that others in their community are highly coordinated with them they bare less of the cost enforcing a particular moral rule and benefit from other's compliance. If a participant believes that others will have the same moral evaluation of a situation it means that he is highly coordinated. All responses were reversed scored.

#### The multigroup ethnic identity measure-revised (MEIM-R)

The *Multigroup Ethnic Identity Measure-Revised* (MEIM-R) (Phinney, 1992; Roberts et al., 1999) measures two factors of ethnic identification, *ethnic identity search* and *ethnic commitment*, on a 4-point scale from 1 (*strongly disagree*) to 4 (*strongly agree*). *Ethnic identity search* includes five items that measure the extent to which participants seek information and experiences relevant to one's ethnicity, such as reading and talking to people, learning cultural practices, and attending cultural events (e.g., *I have spent time trying to find out more about my ethnic group, such as its history, traditions, and customs*; *I think a lot about how my life will be affected by my ethnic group membership*). *Ethnic commitment* comprises seven items that measure an individual's strong attachment and a personal investment in his/her ethnic group (e.g., *I have a lot of pride in my ethnic group*; *I feel a strong attachment toward my own ethnic group*). Items were averaged to create a composite score. Cronbach's α was 0.78 for *ethnic identity search* and 0.92 for *ethnic commitment*.

#### Relational mobility scale

The *Relational Mobility Scale* (Yuki et al., 2007) is a measure of perceived opportunities to form new relationships in one's social environment. It should be noted that these scores reflect a participant's perception that others in his/her social world are able to freely form new relationships, which may differ from the participant's actual ability to freely associate with others. What is more important for our analysis is not the actual costs involved in finding new social relationships, but how the participant understands his/her social world and perceives the potential exit costs. Participants are asked to rate 12 items on a 1 (*strongly disagree*) to 7 (*strongly agree*) scale regarding the mobility of others in their immediate environment (e.g., *If they did not like their current groups, they would leave for better ones*; *It is easy for them to meet new people*; *They have many chances to get to know other people*). Items were averaged to create a composite score. Cronbach's α was 0.82 for the scale. It is noteworthy that relational mobility is not significantly higher for our urban participants than it is for our rural participants (*p* = 0.12).

#### Guilt measure

Participants were asked to judge how guilty the agent who acted wrongfully in the scenarios would feel. Six items from the *Harder Personal Feelings Questionnaire Guilt Scale* were adopted to measure that perceived guilt (Harder and Lewis, 1987). Participants were asked: *Do you think that* [the protagonist] *would experience any of the following feelings?* (*e.g*., mild guilt, remorse, regret). Five filler items were also included in the assessment of potential emotional responses (e.g., enjoyment, rage, depression). Responses were measured on a 0 (*they would not experience the feeling*) to 4 scale (*they would experience the feeling very strongly*) and then averaged to create composite scores for both same and opposing coalition scenarios. Cronbach's α was 0.91 for the within-coalition guilt scale and 0.92 for the without-coalition guilt scale.

### Procedure

We investigate whether our urban and rural communities differ with regard to their moral evaluations of wrongdoing involving characters belonging to either the same social unit (*within-coalition*) or not (*without-coalition*) and whether individual variables account for these differences in moral judgments. Participants from Zadar and Benkovac areas were randomly assigned to one of two survey packets. Every participant went over either a *within* or *without* version of every moral vignette, for a total of two *within* and two *without* conditions for each participant (see Table 3). The design included a 2 (*within*-/*without*-coalition) × 2 (urban/rural) between-subjects design. Thus, although each participant rated *within* and *without*-coalition vignettes, they never rated the same vignette for both *within* and *without* condition.

**Table 3:** Presentation order of survey vignettes.

**Vignette**	**Set A**	**Set B**
1. Gambles	*Filler*	*Filler*
2. Stuck on road	*Within*-Coalition	*Without*-Coalition
3. Jumps bridge	*Filler*	*Filler*
4. Punch neighbor	*Without*-Coalition	*Within*-Coalition
5. Drinks too much	*Filler*	*Filler*
6. Gossip neighbor	*Within*-Coalition	*Without*-Coalition
7. Marijuana	*Filler*	*Filler*
8. Wallet	*Without*-Coalition	*Within*-Coalition

*Sets A and B were also counterbalanced for Chatic/Lamak labels and order effects*.

After having given their consent, participants were handed a survey packet that consisted of four main sections. All survey materials were translated into Serbo-Croatian by a certified translator, back translated by a second translator, and inconsistencies reconciled. Participants were asked to read the following general information: *The Chatic people and the Lamak are two groups that live together in the same area. Individuals of the two groups interact from time to time, but not as often as they do with members of their community. The following interactions occurred by the center of the town*. As a comprehension check after reading the introduction, participants were asked to respond to the following question: *Before continuing, please tell us whether the members of the two communities interact more with individuals of their own community or those outside of their community?*

In the first section of the survey, participants were asked to read descriptions of social situations that occurred in a fictitious location [e.g., *A Lamak man is driving around completing his errands for the day and sees a Chatic man stuck on the side of the road. He does not help the man and continues driving*.] and to rate the behavior of the agent wrongfully acting on a seven point Likert-type scale from 1 (*extremely bad*) to 7 (*extremely good*). Participants randomly received one of four versions of the survey, which were counterbalanced for order of the moral scenarios and the within/without-coalition conditions (see *supplementary materials*). The group member names (Lamak or Chatic) were randomized. Participants were then asked to respond to the guilt measure. Participants next evaluated what other people would be likely to think about the protagonist's behavior (*What would people think of the man if they knew what he had done? Would they think he is a good or bad person?*) on a 1 (*extremely bad*) to 7 (*extremely good*) Likert-type scale. Once this section had been completed, participants filled out the *Relational Mobility Scale* and the MEIM-R. Lastly, participants completed a brief demographics form that assessed age (categorized 1 = *18–24* to 16 = *95*+), sex, income (categorized 1 = < *3,500 kuna/*~*$560 a month* to 11 = *over 17,000 kuna/*~*$2,750 a month*), education (categorized 1 = *no schooling* to 13 = *doctorate degree*), and marital status (collapsed to unmarried/married for analysis; see supplementary material “Demographics” for additional information).

## Results

### Do ratings differ for *without-* and *within-coalition* moral evaluations between urban and rural environments?

Given that the primary areas of mobilization at the onset of the Homeland War were in rural areas, we first wanted to see if our urban and rural participants differed in their moral evaluations. An initial MANOVA was run with *without-* and *within-coalition* moral evaluations as dependent variables, and *urban-rural* as the independent variable. Statistical assumptions for MANOVA were met. There was no multicollinearity, as assessed by Pearson correlations (Tables 4, 5). One participant was removed as a multivariate outlier from the analysis. There were no other multivariate outliers in the data, as assessed by Mahalanobis distance (*p* > 0.001). There was homogeneity of variance-covariance matrices, as assessed by Box's test of equality of covariance matrices (*p* = 0.002) and homogeneity of variances, as assessed by Levene's Test of Homogeneity of Variance (*p* > 0.05). All assumptions were met for further analyses.

**Table 4:** Descriptive Statistics.

**Variable**	***N***	**Minimum**	**Maximum**	**Mean**	**Std. Deviation**
Urban/Rural	105	0.00	1.00	0.53	0.50
Age^*^	105	1.00	14.00	4.54	2.93
Education^*^	102	2.00	13.00	6.32	2.70
Income^*^	94	1.00	10.00	1.99	1.29
Marital status^*^	105	0.00	1.00	0.70	0.46
Sex	105	0.00	1.00	0.57	0.50
Coordination	105	−12.00	0.00	−3.17	2.76
Relational mobility	105	2.80	7.00	4.92	0.99
Ethnic commitment	105	2.14	4.00	3.15	0.48
Ethnic search	105	1.60	4.00	2.81	0.54
Within-coalition moral evaluations	105	4.50	7.00	6.27	0.61
Without-coalition moral evaluations	105	4.50	7.00	6.17	0.68
Within guilt	103	0.00	2.83	0.89	0.77
Without guilt	103	0.00	2.83	0.92	0.78

*Marriage status was collapsed to unmarried/married for analysis*.

* *see supplementary material ‘Demographics’ for categorical breakout*.

**Table 5:** Pearson correlations.

**Variable**	**1**	**2**	**3**	**4**	**5**	**6**	**7**	**8**	**9**	**10**	**11**	**12**	**13**
1. Age													
2. Income	−0.02												
3. Edu.	−0.18	0.45^*^											
4. Mar.	0.33^*^	0.11	−0.03										
5. Sex	0.04	−0.15	−0.13	0.05									
6. U/R	0.27^*^	−0.26^*^	−0.58^*^	0.17	0.12								
7. Coord.	0.18	−0.05	−0.18	0.01	0.01	0.26^*^							
8. Mobil.	0.02	−0.18	−0.15	0.07	0.07	0.15	0.17						
9. E.C.	0.14	−0.10	−0.12	0.17	0.10	0.37^*^	0.03	0.07					
10. E.S.	−0.01	0.03	0.01	0.16	0.05	0.18	0.08	−0.02	0.64^*^				
11. WI.G	−0.22^*^	0.36^*^	0.24^*^	0.06	−0.10	−0.29^*^	−0.29^*^	−0.13	0.07	0.09			
12. WO.G	−0.21^*^	0.23^*^	0.25^*^	0.08	−0.12	−0.36^*^	−0.30^*^	−0.08	−0.02	−0.04	0.70^*^		
13. WI.M	0.23^*^	−0.11	−0.08	0.19	0.16	0.25^*^	0.22^*^	0.21^*^	0.11	0.08	−0.23^*^	−0.31^*^	
14. WO.M	0.21^*^	−0.14	−0.11	0.15	0.11	0.22^*^	0.41^*^	0.37^*^	0.19	0.02	−0.33^*^	−0.25^*^	0.60^*^

* *p < 0.05*;

*U/R,Urban/Rural; Coord, Moral coordination; Mobil, Relational Mobility; E.C., Ethnic commitment; E.S., Ethnic search; WI.G, Within guilt; WO.G, Without Guilt; WI.M, Within moral evaluation; WO.M, Without moral evaluation*.

There was a statistically significant difference between the *urban*-*rural* variable on the combined dependent variables [*F*_(2, 101)_ = 4.20, *p* = 0.018; Wilks' Λ = 0.923; partial η^2^ = 0.08]. There was a statistically significant difference in *without-coalition* moral evaluations [*F*_(1, 102)_ = 6.65, *p* = 0.011; partial η^2^ = 0.061] between *urban-rural* (*M*_urban_ = 6.01, *SD*_urban_ = 0.66; *M*_rural_ = 6.33, *SD*_rural_ = 0.63). There was also a statistically significant difference in *within-coalition* moral evaluations [*F*_(1, 102)_ = 6.92, *p* = 0.010; partial η^2^ = 0.063] between *urban*-*rural* (*M*_urban_ = 6.10, *SD*_urban_ = 0.55; *M*_rural_ = 6.40, *SD*_rural_ = 0.63).

We wanted to assess whether our independent variables of interest, specifically *moral coordination* and *social mobility*, account for the difference between the urban and rural moral evaluations. A second MANOVA examined *without-* and *within-coalition* moral evaluations as dependent variables and *urban/rural, ethnic identity search, ethnic commitment, relational mobility, moral coordination, age, sex, income, education*, and *marital status* (married or unmarried) as independent variables. All variables were mean centered and missing values were replaced with the mean. Statistical assumptions for MANOVA were satisfied. The overall model is statistically significant [*F*_(22, 182)_ = 2.49, *p* < 0.001; Wilks' Λ = 0.591]. However, there was no longer a statistically significant difference between *urban-rural* on the combined dependent variables [*F*_(2, 91)_ = 1.42, *p* = 0.247; Wilks' Λ = 0.970; partial η^2^ = 0.03]. There was a statistically significant effect of *relational mobility* [*F*_(2, 91)_ = 3.45, *p* = 0.036; Wilks' Λ = 0.929; partial η^2^ = 0.071] and *moral coordination* [*F*_(2, 91)_ = 10.89, *p* < 0.001; Wilks' Λ = 0.807; partial η^2^ = 0.193] on the combined dependent variables. No other variables were statistically significant.

### What variables are predictive of *without-* and *within-coalition* moral evaluations?

With the effect of *urban-rural* significantly reduced by our other variables of interest, we were interested to see how our independent variables predict moral evaluations. Hence, we conducted a multivariate multiple regression in which the *without-coalition* and *within-coalition* moral evaluations were the two dependent variables, retaining *urban-rural, ethnic identity search, ethnic commitment, relational mobility, moral coordination* as predictor variables, while controlling for *age, sex, income, education*, and *marital status* (married or unmarried). The effect of the survey set to which participants responded was controlled for by adding a dummy variable in the multivariate regression.

The effect size of the multivariate regression reached significance for the *without-coalition* moral evaluations [*R*^2^ = 0.35, *F*_(11, 92)_ = 4.46, *p* < 0.001], but only came close to significance for the *within-coalition* moral evaluations (*p* = 0.052) with no variables reaching statistical significance (see Table 6). *Moral coordination* [*b* = 0.10, β = 0.40, *t*_(92)_ = 4.76, *p* < 0.001, 95% CI (0.05,0.14)] and *relational mobility* [*b* = 0.16, β = 0.23, *t*_(92)_ = 2.84, *p* = 0.010, 95% CI (0.04,0.27)] were significant predictors of *without-coalition* moral evaluations. No other variables reached significance, however *ethnic commitment* did approach statistical significance for *without-coalition* moral evaluations (*p* = 0.06), but not for *within-coalition* moral evaluations (*p* = 0.76). It is noteworthy that ethnic commitment is higher for rural than urban participants [*F*_(1, 103)_ = 16.74, *p* < 0.001].

**Table 6:** Multivariate multiple regression of moral evaluations.

**Variable**	**Model**	***b***	***SE(b)***	**β**	***t***	***p***
Intercept	*Within*	5.97	0.16		36.83^***^	<0.001
	*Without*	5.95	0.16		38.10^***^	<0.001
Vignette Control	*Within*	0.01	0.13	0.01	0.08	0.938
	*Without*	0.01	0.12	0.01	0.08	0.939
Urban/Rural	*Within*	0.25	0.16	0.21	1.57	0.119
	*Without*	0.06	0.15	0.04	0.38	0.704
Sex	*Within*	0.14	0.12	0.12	1.21	0.228
	*Without*	0.12	0.11	0.09	1.08	0.283
Married	*Within*	0.13	0.14	0.10	0.97	0.334
	*Without*	0.17	0.13	0.12	1.30	0.198
Age	*Within*	0.03	0.02	0.15	1.45	0.149
	*Without*	0.01	0.02	0.05	0.50	0.617
Income	*Within*	−0.05	0.06	−0.11	−0.96	0.339
	*Without*	−0.04	0.05	−0.07	−0.70	0.484
Education	*Within*	0.04	0.03	0.18	1.40	0.165
	*Without*	0.02	0.03	0.09	0.81	0.418
Ethnic Commitment	*Within*	−0.05	0.17	−0.04	−0.33	0.751
	*Without*	0.30	0.17	0.22	1.78	0.078
Ethnic Search	*Within*	0.05	0.14	0.04	0.32	0.750
	*Without*	−0.21	0.14	−0.17	−1.48	0.142
Relational Mobility	*Within*	0.10	0.06	0.15	1.56	0.122
	*Without*	0.16	0.06	0.23	2.64^**^	0.010
Moral Coordination	*Within*	0.03	0.02	0.13	1.26	0.209
	*Without*	0.10	0.02	0.40	4.44^***^	<0.001

* p < 0.05,

** *p < 0.01*,

*** *p < 0.001*.

*Within-coalition: F_(11, 93)_ = 1.79, p = 0.07, R^2^ = 0.17*.

*Without-coalition: F_(11, 93)_ = 5.06, p < 0.0001, R^2^ = 0.37*.

**Figure 2:**
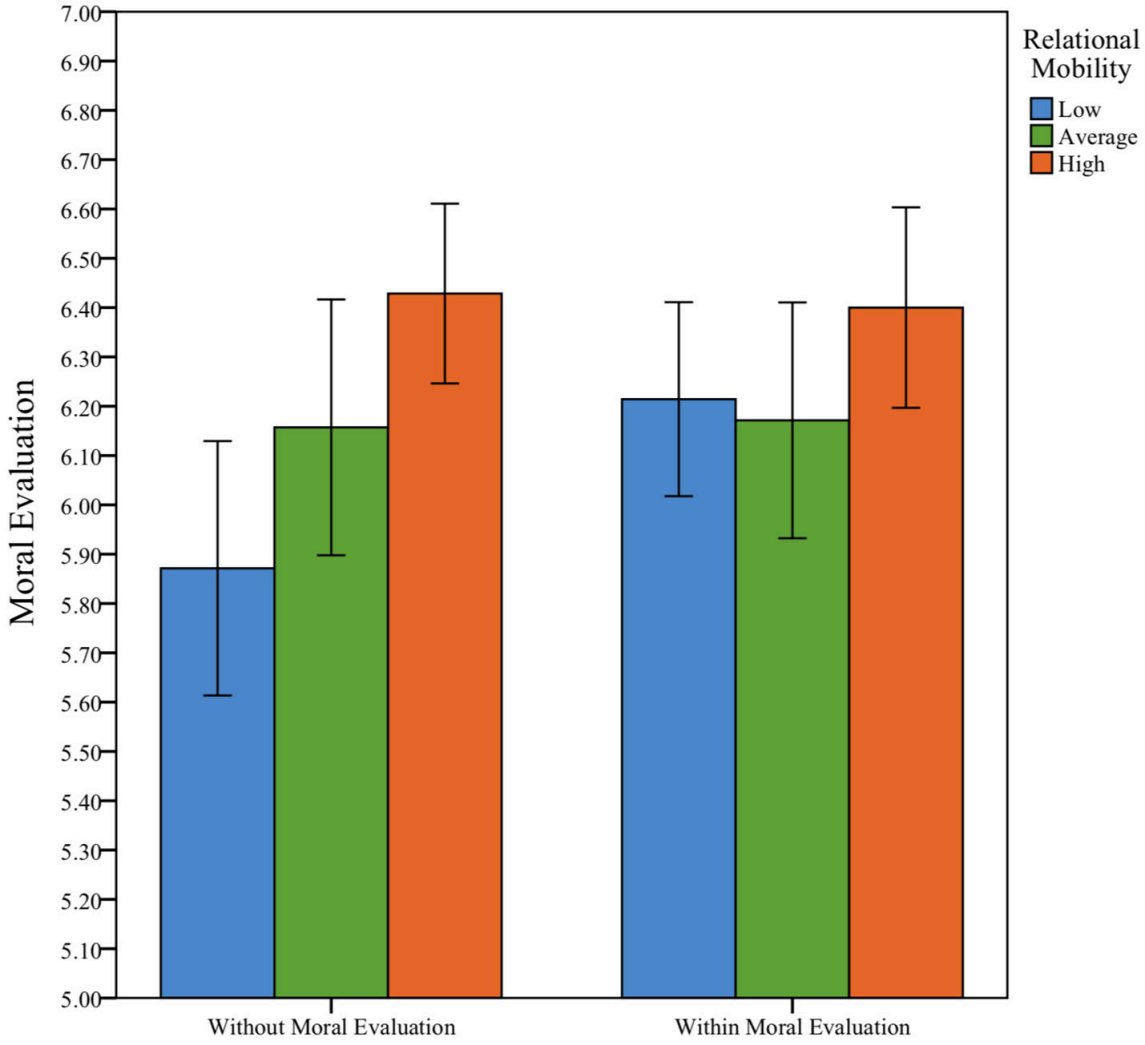
Mean moral evaluations for low, average, and high levels of relational mobility.

**Figure 3:**
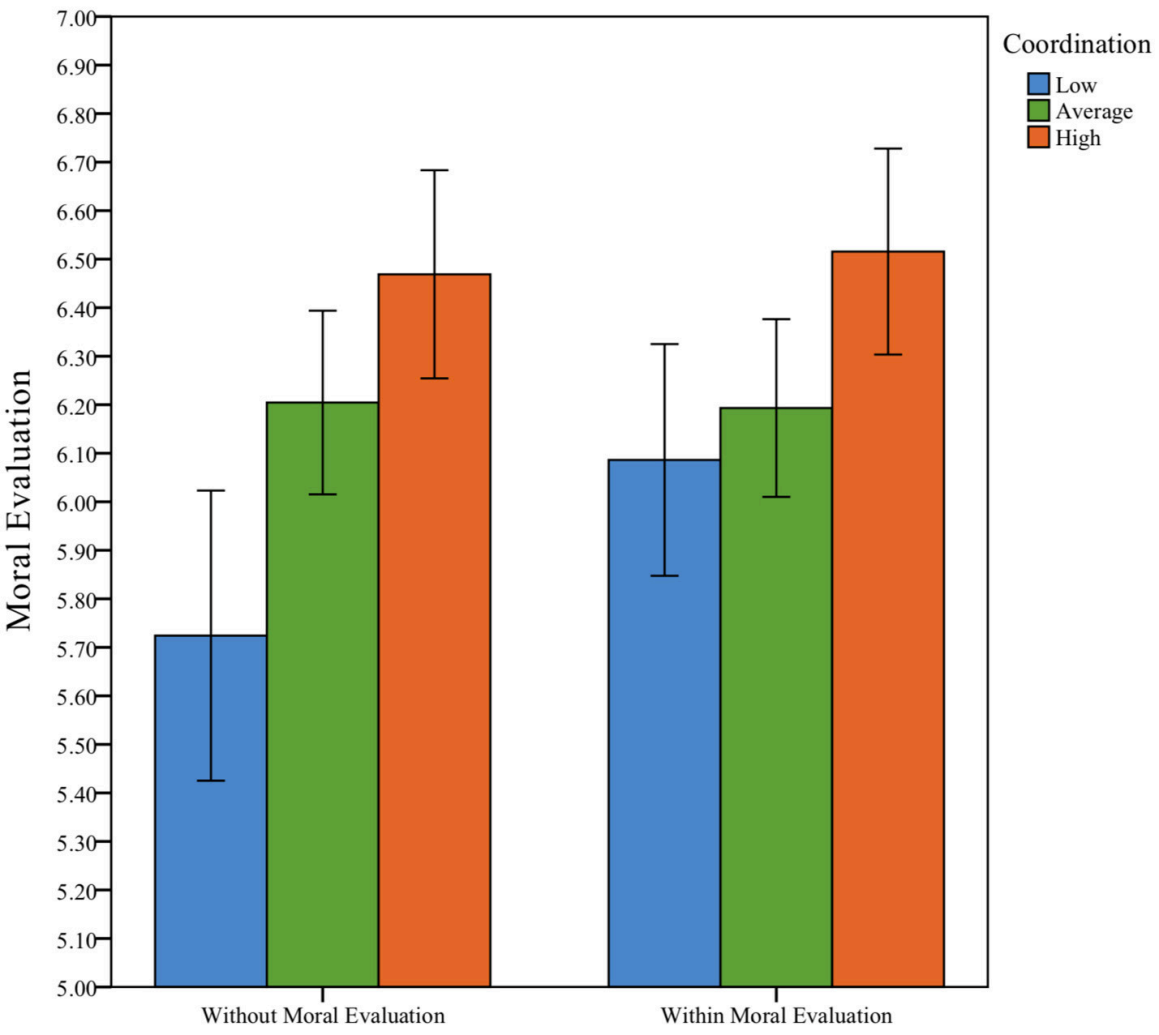
Mean moral evaluations for low, medium, and high levels of coordination.

To more clearly visualize the relationship between moral evaluation scores, and *moral coordination* and *relational mobility* we ranked cases of the two variables into high, average, and low scores (see Figures 2, 3). As can be seen in the figures, lower *relational mobility* and low *moral coordination* scores are associated with lower *without-coalition* moral evaluations, but not *within-coalition* moral evaluations. Indeed, the participants low in *relational mobility* and *moral coordination* appear to be driving the observed effect in the regression.

### Does coordination mediate the relationship between urban/rural and moral evaluations?

**Figure 4:**
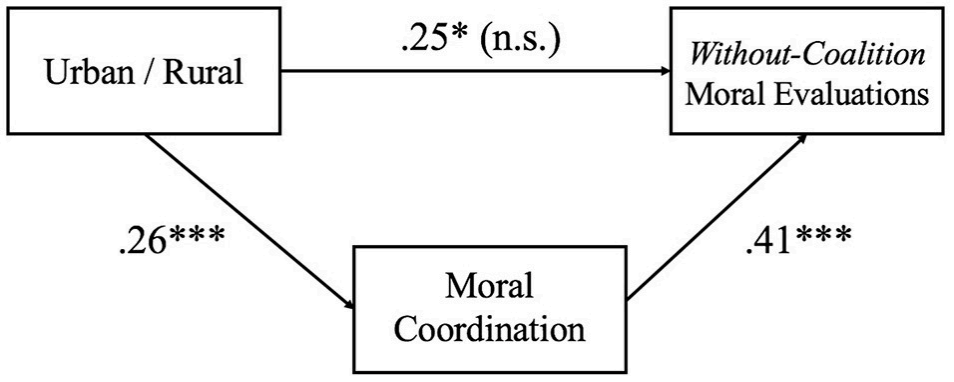
Relationship between urban/rural and without-coalition moral evaluations as mediated by moral coordination. Standardized regression coefficients for the relationship between urban/rural and *without coalition* moral evaluation as mediated by moral coordination. ^*^p < 0.05, ^***^p < 0.001.

We rely on a regression analysis to investigate the hypothesis that moral coordination mediates the effect of *urban-rural* on *within-coalition* and *without-coalition* moral evaluations (see Figure 4). Moral coordination mediates the effect of *urban-rural* on *without-coalition* moral evaluations, but not *within-coalition* moral evaluations. *Urban-rural* was a significant predictor of *without-coalition* moral evaluations [*b* = 0.33, β = 0.25, *SE* = 0.13, *t*_(102)_ = 2.58, *p* = 0.011, 95% CI (0.08,0.58)] and moral coordination was a significant predictor of *without-coalition* moral evaluations [*b* = 0.10, β = 0.41, *SE* = 0.02, *t*_(101)_ = 4.50, *p* < 0.001, 95% CI (0.06,0.14)]. *Urban-rural* was no longer a significant predictor of *without-coalition* moral evaluations after controlling for the mediator *moral coordination* [*b* = 0.19, β = 0.14, *SE* = 0.12, *t*_(101)_ = 1.57, *p* = 0.120, 95% CI (−0.05,0.43)], supporting the mediation hypothesis. Approximately 22% of the variance in *without-coalition* moral evaluations was accounted for by the predictors (*R*^2^ = 0.218). The indirect effect was tested using a bootstrap estimation approach with 1,000 samples. These results indicated that the indirect coefficient was significant, [*b* = 0.14, *SE* = 0.06, *p* = 0.024, 95% CI = (0.05,0.28)].

### Do ratings differ for *without-* and *within-coalition* guilt evaluations between the urban and rural environments?

As previously discussed, Sznycer et al. (2012) found that proneness to shame increases in environments where the cost of being sanctioned for a violation is great. Given their functional role in maintaining moral coordination, the proneness to feel guilty should also be sensitive to the dynamics of a social environment. We first wanted to see if our urban and rural participants differed in their guilt evaluations. We ran an initial MANOVA with the *without-* and *within-coalition* guilt evaluations as dependent variables and *urban-rural* as the independent variable. Statistical assumptions for MANOVA were met. There was no multicollinearity, as assessed by Pearson correlations. One participant was removed as a multivariate outlier from the analysis. There were no other multivariate outliers in the data, as assessed by Mahalanobis distance (*p* > 0.001). There was homogeneity of variance-covariance matrices, as assessed by Box's test of equality of covariance matrices (*p* = 0.093) and homogeneity of variances, as assessed by Levene's Test of Homogeneity of Variance (*p* > 0.342).

There was a statistically significant difference between the *urban-rural* variable on the combined dependent variables [*F*_(2, 100)_ = 7.95, *p* < 0.001; Wilks' Λ = 0.863; partial η^2^ = 0.142]. There was a statistically significant difference in *without-coalition* guilt evaluations [*F*_(1, 101)_ = 16.58, *p* < 0.001; partial η^2^ = 0.141] between *urban-rural* (*M*_urban_ = 1.23, *SD*_urban_ = 0.72; *M*_rural_ = 0.64, *SD*_rural_ = 0.72). There was also a statistically significant difference in *within-coalition* moral evaluations [*F*_(1, 101)_ = 9.33, *p* = 0.003; partial η^2^ = 0.085] between *urban-rural* (*M*_urban_ = 1.13, *SD*_urban_ = 0.67; *M*_rural_ = 0.69, *SD*_rural_ = 0.79).

We wanted to see if our independent variables might account for the difference between the urban and rural guilt evaluations. We ran a second MANOVA with the *without-* and *within-coalition* guilt evaluations as dependent variables and *urban-rural, ethnic identity search, ethnic commitment, relational mobility, moral coordination, within-* and *without-coalition moral evaluations, age, sex, income, education*, and *marital status* (married or unmarried) as independent variables. All variables were mean centered and missing values were replaced with the mean.

Results of the second MANOVA indicate that the overall model is statistically significant [*F*_(26, 176)_ = 2.30, *p* < 0.001; Wilks' Λ = 0.557]. There was a statistically significant effect of *urban-rural* [*F*_(2, 88)_ = 3.15, *p* = 0.048; Wilks' Λ = 0.933; partial η^2^ = 0.067] and *income* [*F*_(2, 88)_ = 5.94, *p* = 0.004; Wilks' Λ = 0.881; partial η^2^ = 0.119] on the combined dependent variables. There was a statistically significant interaction for *urban-rural* with *relational mobility*, and *within-* and *without*-*coalition* moral evaluations, indicating that homogeneity of regression slopes was violated.

To examine how our urban and rural models differed, we split our sample into urban and rural and conducted multivariate multiple regression in which the *without-coalition* guilt and *within-coalition* guilt evaluations were the two dependent variables, retaining *ethnic identity search, ethnic commitment, relational mobility, moral coordination, within-* and *without*-*coalition* moral evaluations as predictors, while controlling for *age, sex, income, education*, and *marital status* (married or unmarried). We controlled for differences in the vignette set, by including a control variable in the multivariate regression (note that only statistically significant variables are shown in Tables 7, 8 for clarity).

**Table 7:** Multivariate multiple regression of guilt evaluations for urban participants.

**Variable**	**Model**	***b***	***SE(b)***	**β**	***t***	***p***
Age	*Within Guilt*	−0.08	0.04	−0.33	−2.13^*^	0.040
	*Without Guilt*	−0.05	0.04	−0.18	−1.09	0.281
Income	*Within Guilt*	0.18	0.06	0.40	2.98^**^	0.005
	*Without Guilt*	0.05	0.07	0.10	0.70	0.490
Relational Mobility	*Within Guilt*	0.29	0.15	0.32	1.96	0.058
	*Without Guilt*	0.52	0.17	0.53	3.05^**^	0.004

* *p < 0.05*,

** *p < 0.01*,

*** p < 0.001.

*Within-guilt: F_(12, 35)_ = 2.92, p = 0.007, R^2^ = 0.50*.

*Without-guilt: F_(12, 35)_ = 2.19, p = 0.036, R^2^ = 0.23*.

*Models include additional variables not shown in the table for clarity*.

**Table 8:** Multivariate multiple regression of guilt evaluations for rural participants.

**Variable**	**Model**	***b***	***SE(b)***	**β**	***t***	***p***
*Within-Coalition*	*Within Guilt*	0.57	0.25	0.41	2.28^*^	0.028
Moral Evaluation	*Without Guilt*	0.29	0.25	0.23	1.17	0.247
*Without-Coalition*	*Within Guilt*	−0.86	0.23	−0.72	−3.74^**^	0.001
Moral Evaluation	*Without Guilt*	−0.61	0.23	−0.56	−2.73^*^	0.009

* *p < 0.05*,

** *p < 0.01*,

***p < 0.001.

*Within-guilt: F_(12, 42)_ = 3.36, p = 0.002, R^2^ = 0.49*.

*Without-guilt: F_(12, 42)_ = 2.41, p = 0.018, R^2^ = 0.41*.

*Models include additional variables not shown in the table for clarity*.

### What variables are predictive of perceptions of protagonist's guilt?

Interestingly, for our urban participants, *within- and without-coalition* moral evaluations were not significant predictors of *within- and without-coalition* guilt evaluations. Increased *relational mobility* was a predictor of *without-*guilt evaluations [*b* = 0.52, β = 0.53, *t*_(35)_ = 3.05, *p* = 0.004, 95% CI (0.21,0.88)], however *within-*guilt only comes close to significance (see Table 7). *Income* was also positively associated with increased *within-*guilt [*b* = 0.18, β = 0.40, *t*_(35)_ = 2.98, *p* = 0.005, 95% CI (0.06,0.31)] whereas *age* was associated with decreased *within-*guilt evaluations [*b* = −0.08, β = −0.33, *t*_(35)_ = −2.13, *p* = 0.040, 95% CI (−0.15, −0.01)]. No other variables were statistically significant.

For our rural participants, harsher *within-coalition* moral evaluations predicted increased *within-coalition* guilt evaluations [*b* = 0.57, β = 0.41, *t*_(42)_ = 2.28, *p* = 0.028, 95% CI (0.21, 1.03)] (see Table 8). Our rural participants who harshly condemn within-coalition harms are more likely to believe that the protagonist will experience guilt for their actions. In contrast, harsher *without-coalition* moral evaluations was a predictor of both lower *within-coalition* guilt evaluations [*b* = −0.86, β = −0.72, *t*_(42)_ = −3.74, *p* = 0.001, 95% CI (−1.33, −0.46)] and lower *without-coalition* guilt evaluations [*b* = −0.61, β = −0.56, *t*_(42)_ = −2.73, *p* = 0.009, 95% CI (−0.88, −0.02)]. No other variables were statistically significant. These effects are interesting for they indicate that participants who condemn harshly inter-coalitional harm, and therefore are also sensitive to the coalitional dimension of the moral scenarios, may be less likely to believe that the protagonist will experience guilt for their actions regardless of social affiliation.

### Is there an interaction between relational mobility and urban/rural?

We had no strong prediction about perceptions of third-party guilt, but since our urban participants rely more upon an open social world, they should be more inclined to ascribe a moral standing to outsiders than others more entrenched in a smaller social environment. In line with this reasoning, we find differences between how urbanites and rural residents conceive of protagonist guilt given their degree of relational mobility. *Without-* and *within-coalition* guilt evaluations were regressed on *relational mobility* and *urban/rural*. There was a statistically significant interaction for both models. To model the interaction between *relational mobility* and *urban/rural* when explaining *without-* and *within-coalition* guilt we used the program PROCESS (Hayes, 2013).

*Without- coalition* guilt evaluations and *within-coalition* guilt evaluations were regressed on *urban/rural* with *relational mobility* as a moderating variable. The overall regression for *without-coalition* guilt evaluations [*R*^2^ = 0.27, *F*_(3, 100)_ = 12.14, *p* < 0.001] and *within-coalition* guilt evaluations [*R*^2^ = 0.21, *F*_(3, 100)_ = 8.81, *p* < 0.001] were both statistically significant. *Urban/rural* [*b* = −0.56, β = −0.36, *t*_(100)_ = −4.14, *p* < 0.001, 95% CI (−83, −0.29)] and *relational mobility* [*b* = 0.48, β = 0.61, *t*_(100)_ = 3.61, *p* < 0.001, 95% CI (0.21,0.75)] were statistically significant predictors of *without-coalition* guilt evaluations. *Urban/rural* [*b* = −0.44, β = −0.27, *t*_(100)_ = −3.14, *p* = 0.002, 95% CI (−0.72, −0.16)] and *relational mobility* [*b* = 0.39, β = 0.50, *t*_(100)_ = 2.83, *p* = 0.006, 95% CI (0.12,0.67)] were also statistically significant predictors of *within-coalition* guilt evaluations. The interaction of *relational mobility* and *urban/rural* was also statistically significant for *without-coalition* guilt evaluations (*b* = −0.68, β = −0.737, *t*_(100)_ = −4.38, *p* < 0.001, 95% CI (−0.99, −0.37)] and *within-coalition* guilt evaluations [*b* = −0.62, β = −0.68, *t*_(100)_ = −3.86, *p* < 0.001, 95% CI (−0.94, −0.30)].

**Figure 5:**
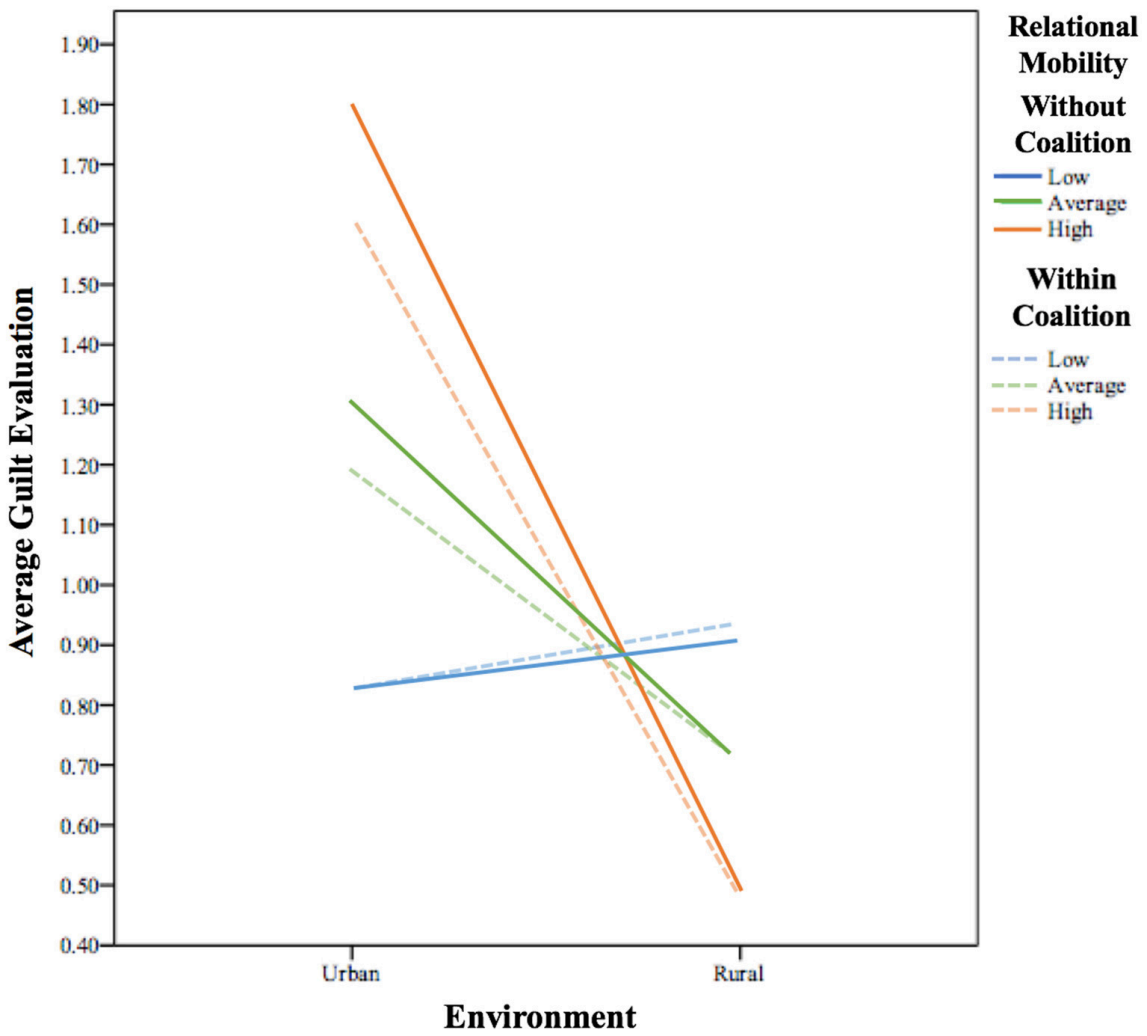
The Effect of urban/rural on guilt evaluations at low, average, and high relational mobility.

We examined the conditional effect of *urban/rural* on *without-coalition* guilt evaluations at (1) one standard deviation below the mean of *relational mobility*, (2) the mean, and (3) one standard deviation above the mean. For low *relational mobility*, there is no relationship between *urban/rural* and *without-coalition* moral evaluations (*p* = 0.72). For average *relational mobility*, every unit increase in *urban/rural* gives a −0.61 unit increase in *without-coalition* moral evaluations [*b* = −0.61, *t*_(100)_ = −4.45, *p* < 0.001, 95% CI (−0.87, −0.34)]. For high *relational mobility*, every unit increase in *urban/rural* gives a −1.29 unit increase in *without-coalition* moral evaluations [*b* = −1.29, *t*_(100)_ = −5.96, *p* < 0.001, 95% CI (−1.71, −0.86)] (see Figure 5).

We examined the conditional effect of *urban/rural* on *within-coalition* guilt evaluations at (1) one standard deviation below the mean of *relational mobility*, (2) the mean, and (3) one standard deviation above the mean. For low *relational mobility*, there is no relationship between *urban/rural* and *within-coalition* moral evaluations (*p* = 0.50). For average *relational mobility*, every unit increase in *urban/rural* gives a −0.48 unit increase in *within-coalition* moral evaluations [*b* = −0.48, *t*_(100)_ = −3.41, *p* = 0.001, 95% CI (−0.76, −0.20)]. For high *relational mobility*, every unit increase in *urban/rural* gives a −1.1 unit increase in *within-coalition* moral evaluations (*b* = −1.1, *t*_(100)_ = −4.94, *p* < 0.001, 95% CI (−1.52, −0.66)] (see Figure 5).

## Discussion

We find significant support for the hypothesis (H_1_) that *moral coordination* influences the moral evaluation of harmful actions. *Moral coordination* has a significant mediating role in explaining the relationship between *urban-rural* and moral evaluations. *Moral coordination* seems to matter more for the moral evaluation of interactions between members of two different socio-cultural units than for the evaluation of moral scenarios involving the same coalition members. Previous research has emphasized the importance of coordination in moral condemnation in order to reduce the costs of punishment and potential retaliation (e.g., Boehm, 1993). Indeed, in our study, participants who perceive themselves to be highly coordinated with others in their community are likely to condemn more harshly harmful behavior when this behavior involves members of different coalitions. Our results also support the hypothesis H_2_ that *relational mobility* affects judgments of moral wrongness of behavior. More specifically, H_2.1_ is supported, as *relational mobility* matters more when wrongdoing involves an out-group member. It appears that what drives that effect are the participants who are low in relational mobility, as our highly mobile participants rate similarly wrongful acts perpetrated by either in- or out-group members (H_2.2_). The low mobility participants might be approaching the task with a strong coalitional stance. Given their stronger dependence on a reduced moral circle, low mobility participants might have strong expectations of moral duties for in-group members, hence their stronger outrage in front of cases of wrongdoing involving members of the same coalition. In contrast, individuals who are more mobile deal with a broader social world. The division between in- and out-group should be downplayed. Their ratings of wrongdoing do not differ across conditions.

We find that residents of our rural environments are significantly higher in *ethnic commitment* than our more urban environment (H_3_). This is likely attributable to an increase in exposure to direct violence during the Croatian Homeland War, as well as living in a region that still comprises a significant Serb population. Interestingly, *ethnic commitment* and *ethnic identity search* were not significant predictors of moralization, suggesting that rapid changes in moral accommodations witnessed during periods of ethnic violence may be first the result of an increase in perceived coordination, which might eventually lead to an increase in ethnification, without the latter being the engine of the transformation [see Kuran (1998) for an explanation of the dynamics that might be at play].

We find interesting differences between our urban and rural participants on perceptions of third-party guilt. We find that our highly mobile urban participants are more likely to perceive that perpetrators of harm will experience greater guilt for their actions when the action harms a member of another coalition. When harm is done to an in-group member, guilt evaluation of the same highly mobile urban participants increases, but is only marginally significant. If there is a true discrepancy between evaluations of *within* and *without* wrongdoing, we do not have an explanation. Alternatively, the lack of significance might be due to a problem of sample size. If so, it would mean that the high mobility participants project their expectations of morality evenly on the whole social world, regardless of particular social affiliations. The status of full moral being indeed comes with the ability of feeling guilt. Urbanites, who are younger and have higher income, are more likely to think perpetrators of harm will feel greater guilt when the victim is a member of their own coalition. Being a young, high-income urbanite does not impact one's guilt rating of out-group wrongdoer. We do not have any specific explanation for this difference.

Our rural participants' perceptions of third-party guilt are related to their moral evaluation scores. Rural participants who evaluate interactions between members of different coalitions more harshly are less likely to believe that wrongdoers experience guilt for their actions. However, rural participants who evaluate interactions between members of the same coalition more harshly are more likely to think that these individuals will experience guilt for their actions. The reprehensive nature of the wrongdoing seems to carry over to the assessment of guilt felt in different manners when the wrongdoing involves an in- or an out-group member. Given our sample size, it might be risky to attempt to explain further this effect. It is an interesting finding that should be explored further.

In addition to the overall impact of our measured variables on perceptions of third-party guilt, we find an interesting difference between mobile rural and urban participants in regard to relational mobility. Urban individuals high in relational mobility are more likely to believe that both in- and outgroup perpetrators experience feelings of guilt for their actions afterwards, whereas high relational mobility individuals from rural environments are likely to believe that in- and outgroup perpetrators experience little guilt. Highly mobile, more cosmopolitan individuals (i.e., urban) benefit from an open social world; their guilt judgments match their worldly experience. Highly mobile individuals less dependent on an open social world (i.e., rural) should be more suspicious of unknown others, which should impact their assessment of the moral motivations of those others. Rural and urban participants with low relational mobility report similar levels of perpetrators' guilt (within proximity of the sample mean). Thus, high relational mobility might be associated with a different social strategy in either the rural or the urban environment. Urban high relational mobility would depend on a non-discriminant (i.e., non-coalitional) engagement with the social world, while rural high relational mobility might require some form of ethno-political entrepreneurship.

As previous morality research failed to find a consistently significant effect of demographic factors on moral judgments (Banerjee et al., 2010), we did not originally consider that our demographic variables would play a significant role in moral judgments. However, we find that while our demographic variables did not have a significant effect on moral reasoning, they did have an effect on reasoning about a third-parties' moral dispositions (i.e., guilt). Given that socioeconomic status is implicated in moral reasoning (Bock et al., 1983; Haidt et al., 1993), future research would benefit from considering the effect of socio-economic status as well as perceived relational mobility when making moral judgments. Also, the majority of our sample identified as Croat (98%); it would be interesting to focus on a larger sample including less represented ethnic minorities (i.e., Serbs, Albanians) to investigate whether our results could be replicated. Such additional *minority* segments in our chosen population would plausibly align with the response patterns of our highly coordinated and low relationally mobile participants. Although we define Zadar and the immediate suburbs as distinctly urban, distinct in that from the more rural locality of Benkovac and hinterland, the city has taken in many migrants during and after the homeland war from surrounding rural communities. This introduces a great deal of potential noise into our urban/rural distinction. This might be the reason why we find no significant difference in relational mobility between our two original samples.

The study has revealed some interesting findings, which eventually might help explain the perplexing phenomenon of rapid descent of complex social worlds into chaotic coalitional opposition. Such transformation typically occurs in response to particular economic or social shocks. Major drivers of such transformations might be preexisting networks of coordinated agents, which once proximally extended reach critical levels where new features emerge with specific consequences for the still-unaffiliated individuals. The cost of remaining neutral becoming exorbitant, the fast affiliation of most to well-defined opposing factions would make it appear that what drives the political dynamic might be affiliation to ethnic or national identities.

## Author contributions

MM is responsible for the design, collecting data, analyses, and writing of this paper. PL is responsible for the design, analyses, and writing of this paper.

### Conflict of interest statement

The authors declare that the research was conducted in the absence of any commercial or financial relationships that could be construed as a potential conflict of interest.

## Footnotes

^1^Unless otherwise noted, all data reported is from (Republic of Croatia Bureau of Statistics, 2011).
