# Supplementary material for: Moral Judgments of In-Group and Out-Group Harm in Post-conflict Urban and Rural Croatian Communities
Source: Front Psychol. 2018 Feb 23;9:212. doi: 10.3389/fpsyg.2018.00212 (PMC5829057; doi:10.3389/fpsyg.2018.00212)
Supplement: Supplementary file 1 [file Table1.DOCX]

Supplementary Material

What comes first: Commitment or Coordination?

Moral Judgments of Behavior in Coalitional Environment

Michael Moncrieff, Pierre Lienard*

*** Correspondence:** Pierre Lienard: [Pierre.Lienard@unlv.edu](mailto:Pierre.Lienard@unlv.edu)

**SURVEY MEASURE**

1. **Please read the following information first and answer the two questions that follow:**

**The Chatic people and the Lamak are two groups that live together in the same area. Individuals of the two groups interact from time to time, but not as often as they do with members of their community. The following interactions occurred by the center of the town.**

**Before continuing, please tell us, do members of the two communities interact?**

|  |
| --- |

**2. Please read the description and respond to the question that follows.**

**A ____ man is driving around completing his errands for the day and sees a ____ man stuck on the side of the road. He does not help the man and continues driving.**

| How good or bad is what the man did? Please indicate below. | | | | | | |
| --- | --- | --- | --- | --- | --- | --- |
| Extremely Bad | Bad | Somewhat Bad | Neither Good or Bad | Somewhat Good | Good | Extremely Good |

**A ____ man ends his own life by jumping from a bridge.**

| How good or bad is what the man did? Please indicate below. | | | | | | |
| --- | --- | --- | --- | --- | --- | --- |
| Extremely Bad | Bad | Somewhat Bad | Neither Good or Bad | Somewhat Good | Good | Extremely Good |

**A ____ woman drinks too much alcohol. She falls and injures herself on her way home from a bar.**

| How good or bad is what the woman did? Please indicate below. | | | | | | |
| --- | --- | --- | --- | --- | --- | --- |
| Extremely Bad | Bad | Somewhat Bad | Neither Good or Bad | Somewhat Good | Good | Extremely Good |

**A ____ man gets into an argument with a ____ neighbor and ends up punching the man.**

| How good or bad is what the man did? Please indicate below. | | | | | | |
| --- | --- | --- | --- | --- | --- | --- |
| Extremely Bad | Bad | Somewhat Bad | Neither Good or Bad | Somewhat Good | Good | Extremely Good |

**A ____ boy smokes marijuana for the first time.**

| How good or bad is what the boy did? Please indicate below. | | | | | | |
| --- | --- | --- | --- | --- | --- | --- |
| Extremely Bad | Bad | Somewhat Bad | Neither Good or Bad | Somewhat Good | Good | Extremely Good |

**A ____ woman spreads a rumor about her ____ neighbors stealing from others in the community even though she is unsure if the rumor is true.**

| How good or bad is what the woman did? Please indicate below. | | | | | | |
| --- | --- | --- | --- | --- | --- | --- |
| Extremely Bad | Bad | Somewhat Bad | Neither Good or Bad | Somewhat Good | Good | Extremely Good |

**A ____ man gambles away the last of his personal savings.**

| How good or bad is what the man did? Please indicate below. | | | | | | |
| --- | --- | --- | --- | --- | --- | --- |
| Extremely Bad | Bad | Somewhat Bad | Neither Good or Bad | Somewhat Good | Good | Extremely Good |

**A ____ man finds the wallet of a ____ man on the street. Inside the wallet is an ID and approximately 100 kuna. The man decides to keep the money.**

| How good or bad is what the man did? Please indicate below. | | | | | | |
| --- | --- | --- | --- | --- | --- | --- |
| Extremely Bad | Bad | Somewhat Bad | Neither Good or Bad | Somewhat Good | Good | Extremely Good |

|  |  |  |  |  |  |  |  |  |  |
| --- | --- | --- | --- | --- | --- | --- | --- | --- | --- |

**3. We would now like you to take a few moments to reflect on how the person in each of the situations is likely to feel. Please circle your response.**

| **A ____ man is driving around completing his errands for the day and sees a ____ man stuck on the side of the road. He does not help the man and continues driving.** | | | | | |
| --- | --- | --- | --- | --- | --- |
| Do you think the man in the story would experience any of the following feelings? | | | | | |
|  | Not at all | A little bit | Moderately | Strongly | Very strongly |
| Mild happiness | 0 | 1 | 2 | 3 | 4 |
| Mild guilt | 0 | 1 | 2 | 3 | 4 |
| Feeling they deserve criticism for what they did. | 0 | 1 | 2 | 3 | 4 |
| Sadness | 0 | 1 | 2 | 3 | 4 |
| Regret | 0 | 1 | 2 | 3 | 4 |
| Remorse | 0 | 1 | 2 | 3 | 4 |
| Rage | 0 | 1 | 2 | 3 | 4 |
| Depression | 0 | 1 | 2 | 3 | 4 |
| Enjoyment | 0 | 1 | 2 | 3 | 4 |
| Worry about hurting the other person. | 0 | 1 | 2 | 3 | 4 |
| Intense guilt | 0 | 1 | 2 | 3 | 4 |

| **A ____ man ends his own life by jumping from a bridge.** | | | | | |
| --- | --- | --- | --- | --- | --- |
| Do you think the man in the story would experience any of the following feelings? | | | | | |
|  | Not at all | A little bit | Moderately | Strongly | Very strongly |
| Mild happiness | 0 | 1 | 2 | 3 | 4 |
| Mild guilt | 0 | 1 | 2 | 3 | 4 |
| Feeling they deserve criticism for what they did. | 0 | 1 | 2 | 3 | 4 |
| Sadness | 0 | 1 | 2 | 3 | 4 |
| Regret | 0 | 1 | 2 | 3 | 4 |
| Remorse | 0 | 1 | 2 | 3 | 4 |
| Rage | 0 | 1 | 2 | 3 | 4 |
| Depression | 0 | 1 | 2 | 3 | 4 |
| Enjoyment | 0 | 1 | 2 | 3 | 4 |
| Worry about hurting the other person. | 0 | 1 | 2 | 3 | 4 |
| Intense guilt | 0 | 1 | 2 | 3 | 4 |

| **A ____ woman drinks too much alcohol. She falls and injures herself on her way home from a bar.** | | | | | |
| --- | --- | --- | --- | --- | --- |
| Do you think the woman in the story would experience any of the following feelings? | | | | | |
|  | Not at all | A little bit | Moderately | Strongly | Very strongly |
| Mild happiness | 0 | 1 | 2 | 3 | 4 |
| Mild guilt | 0 | 1 | 2 | 3 | 4 |
| Feeling they deserve criticism for what they did. | 0 | 1 | 2 | 3 | 4 |
| Sadness | 0 | 1 | 2 | 3 | 4 |
| Regret | 0 | 1 | 2 | 3 | 4 |
| Remorse | 0 | 1 | 2 | 3 | 4 |
| Rage | 0 | 1 | 2 | 3 | 4 |
| Depression | 0 | 1 | 2 | 3 | 4 |
| Enjoyment | 0 | 1 | 2 | 3 | 4 |
| Worry about hurting the other person. | 0 | 1 | 2 | 3 | 4 |
| Intense guilt | 0 | 1 | 2 | 3 | 4 |

| **A ____ man gets into an argument with a ____ neighbor and ends up punching the man.** | | | | | |
| --- | --- | --- | --- | --- | --- |
| Do you think the man in the story would experience any of the following feelings? | | | | | |
|  | Not at all | A little bit | Moderately | Strongly | Very strongly |
| Mild happiness | 0 | 1 | 2 | 3 | 4 |
| Mild guilt | 0 | 1 | 2 | 3 | 4 |
| Feeling they deserve criticism for what they did. | 0 | 1 | 2 | 3 | 4 |
| Sadness | 0 | 1 | 2 | 3 | 4 |
| Regret | 0 | 1 | 2 | 3 | 4 |
| Remorse | 0 | 1 | 2 | 3 | 4 |
| Rage | 0 | 1 | 2 | 3 | 4 |
| Depression | 0 | 1 | 2 | 3 | 4 |
| Enjoyment | 0 | 1 | 2 | 3 | 4 |
| Worry about hurting the other person. | 0 | 1 | 2 | 3 | 4 |
| Intense guilt | 0 | 1 | 2 | 3 | 4 |

| **A ____ boy smokes marijuana for the first time.** | | | | | |
| --- | --- | --- | --- | --- | --- |
| Do you think the boy in the story would experience any of the following feelings? | | | | | |
|  | Not at all | A little bit | Moderately | Strongly | Very strongly |
| Mild happiness | 0 | 1 | 2 | 3 | 4 |
| Mild guilt | 0 | 1 | 2 | 3 | 4 |
| Feeling they deserve criticism for what they did. | 0 | 1 | 2 | 3 | 4 |
| Sadness | 0 | 1 | 2 | 3 | 4 |
| Regret | 0 | 1 | 2 | 3 | 4 |
| Remorse | 0 | 1 | 2 | 3 | 4 |
| Rage | 0 | 1 | 2 | 3 | 4 |
| Depression | 0 | 1 | 2 | 3 | 4 |
| Enjoyment | 0 | 1 | 2 | 3 | 4 |
| Worry about hurting the other person. | 0 | 1 | 2 | 3 | 4 |
| Intense guilt | 0 | 1 | 2 | 3 | 4 |

| **A ____ woman spreads a rumor about her ____ neighbors stealing from others in the community though she is unsure if the rumor is true.** | | | | | |
| --- | --- | --- | --- | --- | --- |
| Do you think the woman in the story would experience any of the following feelings? | | | | | |
|  | Not at all | A little bit | Moderately | Strongly | Very strongly |
| Mild happiness | 0 | 1 | 2 | 3 | 4 |
| Mild guilt | 0 | 1 | 2 | 3 | 4 |
| Feeling they deserve criticism for what they did. | 0 | 1 | 2 | 3 | 4 |
| Sadness | 0 | 1 | 2 | 3 | 4 |
| Regret | 0 | 1 | 2 | 3 | 4 |
| Remorse | 0 | 1 | 2 | 3 | 4 |
| Rage | 0 | 1 | 2 | 3 | 4 |
| Depression | 0 | 1 | 2 | 3 | 4 |
| Enjoyment | 0 | 1 | 2 | 3 | 4 |
| Worry about hurting the other person. | 0 | 1 | 2 | 3 | 4 |
| Intense guilt | 0 | 1 | 2 | 3 | 4 |

| **A ____ man gambles away the last of his personal savings.** | | | | | |
| --- | --- | --- | --- | --- | --- |
| Do you think the man in the story would experience any of the following feelings? | | | | | |
|  | Not at all | A little bit | Moderately | Strongly | Very strongly |
| Mild happiness | 0 | 1 | 2 | 3 | 4 |
| Mild guilt | 0 | 1 | 2 | 3 | 4 |
| Feeling they deserve criticism for what they did. | 0 | 1 | 2 | 3 | 4 |
| Sadness | 0 | 1 | 2 | 3 | 4 |
| Regret | 0 | 1 | 2 | 3 | 4 |
| Remorse | 0 | 1 | 2 | 3 | 4 |
| Rage | 0 | 1 | 2 | 3 | 4 |
| Depression | 0 | 1 | 2 | 3 | 4 |
| Enjoyment | 0 | 1 | 2 | 3 | 4 |
| Worry about hurting the other person. | 0 | 1 | 2 | 3 | 4 |
| Intense guilt | 0 | 1 | 2 | 3 | 4 |

| **A ____ man finds the wallet of a ____ man on the street. Inside the wallet is an ID and approximately 100 kuna. The man decides to keep the money.** | | | | | |
| --- | --- | --- | --- | --- | --- |
| Do you think the man in the story would experience any of the following feelings? | | | | | |
|  | Not at all | A little bit | Moderately | Strongly | Very strongly |
| Mild happiness | 0 | 1 | 2 | 3 | 4 |
| Mild guilt | 0 | 1 | 2 | 3 | 4 |
| Feeling they deserve criticism for what they did. | 0 | 1 | 2 | 3 | 4 |
| Sadness | 0 | 1 | 2 | 3 | 4 |
| Regret | 0 | 1 | 2 | 3 | 4 |
| Remorse | 0 | 1 | 2 | 3 | 4 |
| Rage | 0 | 1 | 2 | 3 | 4 |
| Depression | 0 | 1 | 2 | 3 | 4 |
| Enjoyment | 0 | 1 | 2 | 3 | 4 |
| Worry about hurting the other person. | 0 | 1 | 2 | 3 | 4 |
| Intense guilt | 0 | 1 | 2 | 3 | 4 |

|  |  |  |  |  |  |  |  |  |  |
| --- | --- | --- | --- | --- | --- | --- | --- | --- | --- |

**4. Please read the description and respond to the question that follows.**

**A ____ man is driving around completing his errands for the day and sees a ____ man stuck on the side of the road. He does not help the man and continues driving.**

| What would people think of the man if they knew what he had done? Would they think he is a good or bad person? Please indicate below. | | | | | | |
| --- | --- | --- | --- | --- | --- | --- |
| Extremely Bad | Bad | Somewhat Bad | Neither Good or Bad | Somewhat Good | Good | Extremely Good |

**A ____ man ends his own life by jumping from a bridge.**

| What would people think of the man if they knew what he had done? Would they think he is a good or bad person? Please indicate below. | | | | | | |
| --- | --- | --- | --- | --- | --- | --- |
| Extremely Bad | Bad | Somewhat Bad | Neither Good or Bad | Somewhat Good | Good | Extremely Good |

**A ____ woman drinks too much alcohol. She falls and injures herself on her way home from a bar.**

| What would people think of the woman if they knew what she had done? Would they think he is a good or bad person? Please indicate below. | | | | | | |
| --- | --- | --- | --- | --- | --- | --- |
| Extremely Bad | Bad | Somewhat Bad | Neither Good or Bad | Somewhat Good | Good | Extremely Good |

**A ____ man gets into an argument with a ____ neighbor and ends up punching the man.**

| What would people think of the man if they knew what he had done? Would they think he is a good or bad person? Please indicate below. | | | | | | |
| --- | --- | --- | --- | --- | --- | --- |
| Extremely Bad | Bad | Somewhat Bad | Neither Good or Bad | Somewhat Good | Good | Extremely Good |

**A ____ boy smokes marijuana for the first time.**

| What would people think of the boy if they knew what he had done? Would they think he is a good or bad person? Please indicate below. | | | | | | |
| --- | --- | --- | --- | --- | --- | --- |
| Extremely Bad | Bad | Somewhat Bad | Neither Good or Bad | Somewhat Good | Good | Extremely Good |

**A ____ woman spreads a rumor about her ____ neighbors stealing from others in the community even though she is unsure if the rumor is true.**

| What would people think of the woman if they knew what she had done? Would they think he is a good or bad person? Please indicate below. | | | | | | |
| --- | --- | --- | --- | --- | --- | --- |
| Extremely Bad | Bad | Somewhat Bad | Neither Good or Bad | Somewhat Good | Good | Extremely Good |

**A ____ man gambles away the last of his personal savings.**

| What would people think of the man if they knew what he had done? Would they think he is a good or bad person? Please indicate below. | | | | | | |
| --- | --- | --- | --- | --- | --- | --- |
| Extremely Bad | Bad | Somewhat Bad | Neither Good or Bad | Somewhat Good | Good | Extremely Good |

**A ____ man finds the wallet of a ____ man on the street. Inside the wallet is an ID and approximately 100 kuna. The man decides to keep the money.**

| What would people think of the man? Would they think he is a good or bad person? Please indicate below. | | | | | | |
| --- | --- | --- | --- | --- | --- | --- |
| Extremely Bad | Bad | Somewhat Bad | Neither Good or Bad | Somewhat Good | Good | Extremely Good |

|  |  |  |  |  |  |  |  |  |  |
| --- | --- | --- | --- | --- | --- | --- | --- | --- | --- |

**5. Thank you for taking the time to evaluate the behavior of others. We would like to know a bit more about you and your community. Please take a moment to fill out the following information. There are no right or wrong answers.**

**How much do each of the following statements accurately describe the people in the immediate society (your school, workplace, town, neighborhood, etc.) in which you live? Please indicate how true you feel each statement to be for the people around you by checking the appropriate number on the scale provided.**

| 1. **They have many chances to get to know other people.** | | | | | | |
| --- | --- | --- | --- | --- | --- | --- |
| Strongly Disagree | Disagree | Somewhat Disagree | Undecided | Somewhat Agree | Agree | Strongly Agree |
| 1. **It is common for these people to have a conversation with someone they have never met before.** | | | | | | |
| Strongly Disagree | Disagree | Somewhat Disagree | Undecided | Somewhat Agree | Agree | Strongly Agree |
| 1. **They can choose who they interact with.** | | | | | | |
| Strongly Disagree | Disagree | Somewhat Disagree | Undecided | Somewhat Agree | Agree | Strongly Agree |
| 1. **There are few opportunities for these people to form new friendships.** | | | | | | |
| Strongly Disagree | Disagree | Somewhat Disagree | Undecided | Somewhat Agree | Agree | Strongly Agree |
| 1. **It is uncommon for these people to have a conversation with people they have never met before.** | | | | | | |
| Strongly Disagree | Disagree | Somewhat Disagree | Undecided | Somewhat Agree | Agree | Strongly Agree |
| 1. **If they did not like their current groups, they would leave for better ones.** | | | | | | |
| Strongly Disagree | Disagree | Somewhat Disagree | Undecided | Somewhat Agree | Agree | Strongly Agree |
| 1. **It is often the case that they cannot freely choose who they associate with.** | | | | | | |
| Strongly Disagree | Disagree | Somewhat Disagree | Undecided | Somewhat Agree | Agree | Strongly Agree |
| 1. **It is easy for them to meet new people.** | | | | | | |
| Strongly Disagree | Disagree | Somewhat Disagree | Undecided | Somewhat Agree | Agree | Strongly Agree |
| 1. **Even if these people were not completely satisfied with the group they belonged to, they would usually stay with it anyway.** | | | | | | |
| Strongly Disagree | Disagree | Somewhat Disagree | Undecided | Somewhat Agree | Agree | Strongly Agree |
| 1. **These people are able to choose the groups and organizations they belong to.** | | | | | | |
| Strongly Disagree | Disagree | Somewhat Disagree | Undecided | Somewhat Agree | Agree | Strongly Agree |
| 1. **Even if these people were not satisfied with their current relationships, they would often have no choice but to stay with them.** | | | | | | |
| Strongly Disagree | Disagree | Somewhat Disagree | Undecided | Somewhat Agree | Agree | Strongly Agree |
| 1. **Even though they might rather leave, these people often have no choice but to stay in groups they don’t like.** | | | | | | |
| Strongly Disagree | Disagree | Somewhat Disagree | Undecided | Somewhat Agree | Agree | Strongly Agree |

|  |  |  |  |  |  |  |  |  |  |
| --- | --- | --- | --- | --- | --- | --- | --- | --- | --- |

**6. People come from many different countries and cultures, and there are many different words to describe the different back­grounds or ethnic groups that people come from. These questions are about your ethnicity or your ethnic group and how you feel about it or react to it.**

**In terms of ethnic group, I consider myself to be:**

|  | Croat |  | Serb | |
| --- | --- | --- | --- | --- |
|  | Bosniak |  | Other: |  |

| 1. **I have spent time trying to find out more about my ethnic group, such as its history, traditions, and customs.** | | | |
| --- | --- | --- | --- |
| Strongly Agree | Agree | Disagree | Strongly Disagree |
| 1. **I am active in organizations or social groups that include mostly members of my own ethnic group.** | | | |
| Strongly Agree | Agree | Disagree | Strongly Disagree |
| 1. **I have a clear sense of my ethnic background and what it means for me.** | | | |
| Strongly Agree | Agree | Disagree | Strongly Disagree |
| 1. **I think a lot about how my life will be affected by my ethnic group membership.** | | | |
| Strongly Agree | Agree | Disagree | Strongly Disagree |
| 1. **I am happy that I am a member of the group I belong to.** | | | |
| Strongly Agree | Agree | Disagree | Strongly Disagree |
| 1. **I have a strong sense of belonging to my own ethnic group.** | | | |
| Strongly Agree | Agree | Disagree | Strongly Disagree |
| 1. **I understand pretty well what my ethnic group membership means to me.** | | | |
| Strongly Agree | Agree | Disagree | Strongly Disagree |
| 1. **In order to learn more about my ethnic background, I have often talked to other people about my ethnic group.** | | | |
| Strongly Agree | Agree | Disagree | Strongly Disagree |
| 1. **I have a lot of pride in my ethnic group.** | | | |
| Strongly Agree | Agree | Disagree | Strongly Disagree |
| 1. **I participate in cultural practices of my own group, such as special food, music, or customs.** | | | |
| Strongly Agree | Agree | Disagree | Strongly Disagree |
| 1. **I feel a strong attachment towards my own ethnic group.** | | | |
| Strongly Agree | Agree | Disagree | Strongly Disagree |
| 1. **I feel good about my cultural or ethnic background.** | | | |
| Strongly Agree | Agree | Disagree | Strongly Disagree |

|  |  |  |  |  |  |  |  |  |  |
| --- | --- | --- | --- | --- | --- | --- | --- | --- | --- |

**7. Please tell us a little bit about yourself.**

| **1. Age** | **2. Sex** |
| --- | --- |

|  | 18 - 24 |  | 40 - 44 |  | 60 - 64 |  | 80 - 84 |  | Male |
| --- | --- | --- | --- | --- | --- | --- | --- | --- | --- |
|  | 25 - 29 |  | 45 - 49 |  | 65 - 69 |  | 85 - 89 |  | Female |
|  | 30 - 34 |  | 50 - 54 |  | 70 - 74 |  | 90 - 94 |  |  |
|  | 35 - 39 |  | 55 - 59 |  | 75 - 79 |  | 95 + |  |  |

| **3. Religion** | | | | **4. Marital Status** | |
| --- | --- | --- | --- | --- | --- |
|  | | | |  | |
|  | Atheist | |  |  | Single |
|  | Catholic | |  |  | Married / Consensual Union / Partnership |
|  | Orthodox | |  |  | Widowed |
|  | Other: |  |  |  | Divorced |
|  | Prefer not to say | |  |  | Prefer not to say |

| **5. Education** | | | |
| --- | --- | --- | --- |
|  | | | |
|  | No schooling |  | Professional studies lasting less than 3 years |
|  | Basic school 1 – 4 grade |  | Professional studies 3 – 4 years |
|  | Basic school 5 – 8 grade |  | Undergraduate university studies |
|  | Industry and crafts vocational secondary school |  | Post graduate studies |
|  | Technical and related vocational secondary school |  | Masters degree |
|  | Secondary school art |  | Doctorate degree |
|  | Grammar school |  | Prefer not to say |

| **6. Average monthly Income of household** | | | |
| --- | --- | --- | --- |
|  | | | |
|  | Less than 3.500 kuna |  | 11.001 – 12.500 kuna |
|  | 3.501 – 5.000 kuna |  | 12.501 – 14.000 kuna |
|  | 5.001 – 6.500 kuna |  | 14.001 – 15.500 kuna |
|  | 6.501 – 8.000 kuna |  | 15.501 – 17.000 kuna |
|  | 8.001 – 9.500 kuna |  | 17.001 – 18.500 kuna |
|  | 9.501 – 11.000 kuna |  | More than 18.501 kuna |
|  |  |  | Prefer not to say |

| **7. Main source of livelihood (check all that apply)** | | | |
| --- | --- | --- | --- |
|  | | | |
|  | Income from permanent work |  | Property income |
|  | Income from occasional work |  | Social welfare payments |
|  | Income from agriculture |  | Other income |
|  | Old-age pension |  | Periodic support from others |
|  | Other pension |  | Without income |

| **8. Mother tongue** | | | | **9. Number of people living in household** | |
| --- | --- | --- | --- | --- | --- |
|  | | | |  |  |
|  | Croatian |  |  |  |  |
|  | Serbian |  |  |  |  |
|  | Other: |  |  |  |  |

| **10. Occupation** | | **11. Current village / town name** | |
| --- | --- | --- | --- |
|  | |  | |
|  |  |  |  |

| **12. Place and length of residency** | | |
| --- | --- | --- |
|  | Town / City / Village | Length of Times in Years |
| 1 |  |  |
| 2 |  |  |
| 3 |  |  |
| 4 |  |  |
| 5 |  |  |
| 6 |  |  |
| 7 |  |  |
